# Microglia-specific NF-κB signaling is a critical regulator of prion-induced glial inflammation and neuronal loss

**DOI:** 10.1371/journal.ppat.1012582

**Published:** 2025-06-18

**Authors:** Arielle J. D. Hay, Katriana A. Popichak, Genova Mumford, Jifeng Bian, Payton Shirley, Lauren Wolfrath, Michael Eggers, Eric M. Nicholson, Ronald B. Tjalkens, Mark D. Zabel, Julie A. Moreno

**Affiliations:** 1 Prion Research Center, College of Veterinary Medicine and Biomedical Sciences, Colorado State University, Fort Collins, Colorado, United States of America; 2 Department of Microbiology, Immunology and Pathology, College of Veterinary Medicine and Biomedical Sciences, Colorado State University, Fort Collins, Colorado, United States of America; 3 Brain Research Center, Colorado State University, Fort Collins, Colorado, United States of America; 4 Department of Environmental and Radiological Health Sciences, College of Veterinary Medicine and Biomedical Sciences, Colorado State University, Fort Collins, Colorado, United States of America; 5 Virus and Prion Research Unit, National Animal Disease Center, United States Department of Agriculture – Agricultural Research Service, Ames, Iowa, United States of America; Dartmouth College Geisel School of Medicine, UNITED STATES OF AMERICA

## Abstract

Prion diseases are a group of rare and fatal neurodegenerative diseases caused by the cellular prion protein, PrP^C^, misfolding into the infectious form, PrP^Sc^, which forms aggregates in the brain. This leads to activation of glial cells, neuroinflammation, and irreversible neuronal loss, however, the role of glial cells in prion disease pathogenesis and neurotoxicity is poorly understood. Microglia can phagocytose PrP^Sc^, leading to the release of inflammatory signaling molecules, which subsequently induce astrocyte reactivity. Animal models show highly upregulated inflammatory molecules that are a product of the Nuclear Factor-kappa B (NF-κB) signaling pathway, suggesting that this is a key regulator of inflammation in the prion-infected brain. The activation of the IκB kinase complex (IKK) by cellular stress signals is critical for NF-κB-induced transcription of a variety of genes, including pro-inflammatory cytokines and chemokines, and regulators of protein homeostasis and cell survival. However, the contribution of microglial IKK and NF-κB signaling in the prion-infected brain has not been evaluated. Here, we characterize a primary mixed glial cell model containing wild-type (WT) astrocytes and IKK knock-out (KO) microglia. These cultures show a near ablation of microglia compared to WT mixed glial cultures, highlighting the role of IKK in microglial survival and proliferation. We show that, when exposed to prion-infected brain homogenates, NF-κB-associated genes are significantly downregulated, but prion accumulation is significantly increased, in mixed glial cultures containing minimal microglia. Mice with IKK KO microglia show rapid disease progression when intracranially infected with prions, characterized by an increased density of activated microglia and reactive astrocytes, development of spongiosis, and accelerated loss of hippocampal neurons and associated behavioral deficits. These animals display clinical signs of prion disease early and have a 22% shorter life expectancy compared to infected wild-type mice. Intriguingly, PrP^Sc^ accumulation was significantly lower in the brains of terminal animals with IKK KO microglia compared to terminal WT mice, suggesting that accelerated disease is independent of PrP^Sc^ accumulation, highlighting a glial-specific pathology. Together, these findings present a critical role for microglial IKK and NF-κB signaling in host protection against prion disease.

## Author summary

Prion diseases are rare, fatal neurodegenerative diseases caused by the normal prion protein (PrP) in the brain misfolding and aggregating into a version associate with disease, called PrP^Sc^. A hallmark of these diseases in humans and mouse models is inflammation, including activated microglia, reactive astrocytes, and increased levels of inflammatory molecules. Many of these molecules are associated with the Nuclear Factor-kappa B (NF-κB) signaling pathway. This pathway is activated by the IκB kinase complex (IKK) responding to cellular stress signals. The cell types in the prion-infected brain that are responsible for producing these molecules are not fully known. Here, we examine the role of microglia in IKK and NF-κB signaling. Knockout (KO) of IKK effectively dismantles the NF-κB signaling response. We infected mice with IKK KO specific to microglia with prion disease and compared their disease progression to infected mice with normal microglia. Mice with IKK KO microglia succumbed to disease more quickly, and analysis of their brains showed many classic signs of prion disease, except for less PrP^Sc^ accumulation was seen in these mice compared to infected mice with normal microglia. These findings highlight the importance of microglial NF-κB signaling in providing protection to the host in prion infection.

## Introduction

Nuclear Factor-kappa B (NF-κB) signaling in microglia modulates inflammation and protein accumulation in a variety of neurodegenerative diseases [1–3]. However, the role of this signaling pathway in microglia has not been established in prion disease. Prion diseases are rare and fatal neurodegenerative diseases that affect a variety of mammalian species, including humans. The hallmark of these diseases is the misfolding of the cellular prion protein, PrP^C^, to the transmissible form, PrP^Sc^, which acts as a seed and spreads throughout the brain, leading to neuroinflammation and irreversible neurodegeneration. Glial cells, particularly astrocytes and microglia, respond to PrP^Sc^ and release inflammatory molecules that contribute to neuronal death [4–7]. The brains of prion-infected mice have increased cytokines and chemokines that are associated with the NF-κB pathway, some of which are detectable well in advance of clinical signs of disease [8–11]. Previous studies have had conflicting results in whether NF-κB plays a significant role in prion pathogenesis [12,13]. These studies have knocked out critical genes in the NF-κB pathway in astrocytes and neurons, but have largely ignored the role of microglia-specific NF-κB signaling. Microglia are critical for inducing inflammatory activation in astrocytes, which in turn can produce neurotoxic signals that contribute to neurodegeneration [14–17]. Moreover, when exposed to PrP^Sc^ *in vitro*, microglia respond by upregulating genes associated with NF-κB signaling [18].

The NF-κB pathway is responsible for the expression of a large number of genes, including cytokines, chemokines, enzymes, receptors, and regulators of both cell survival and apoptosis. Under normal conditions, NF-κB remains sequestered in the cytoplasm, inhibited from translocating to the nucleus by IκB and associated proteins. The IκB kinase complex (IKK) is activated by a variety of stimuli such as cytokines, growth factors, pathogen-associated molecular patterns (PAMPs) and damage-associated molecular patterns (DAMPs) [19,20]. In prion disease, IKK activation typically occurs through toll-like receptors (TLRs) and nucleotide-binding oligomerization-domain protein-like receptors (NLRs) [20,21]. Activated IKK phosphorylates IκBα, leading to IκBα ubiquitination and degradation by the proteosome. Uninhibited, NF-κB translocates to the nucleus and upregulates pro-inflammatory cytokines.

Here, we demonstrate that microglia-specific knock-out (KO) of IKK leads to near total ablation of microglia in primary astrocyte and microglia co-culture model. This is associated with a downregulation of several NF-κB-related genes when exposed to prion-infected brain homogenates, compared to wild-type (WT) mixed glia. Intriguingly, this is not associated with a decrease in neurotoxicity. We also observed increased accumulation of PrP^Sc^ in glial cultures with limited microglia, suggesting a role of microglia in prion phagocytosis and clearance [22–25]. We further investigated this phenomenon in mice that have myeloid cell-specific IKK KO (including macrophages, monocytes and microglia) [3,26]. These mice were inoculated intracranially with mouse-adapted scrapie prions and assessed for clinical and behavioral changes, survival, glial cell number and morphology, neuronal health and prion accumulation in the brain. Mice with IKK KO microglia succumbed to prion infection 22% faster than WT mice. Compared to wpi-matched WT mice, mice with IKK KO microglia had more glial activation, based on morphology changes and increased density of Iba1 + microglia and GFAP+ astrocytes, more vacuoles in the brain, and a decrease in NeuN+ hippocampal neurons. However, compared to terminal WT mice, terminal mice with IKK KO microglia had less neuronal loss and less detectable PrP^Sc^ throughout the brain. Together, these findings suggest that microglial IKK and NF-κB signaling are critical for host protection against prion infection. Understanding inflammatory signaling pathways and cellular cross-talk in the prion-infected brain is critical for the development of therapeutics.

## Results

### Microglial numbers and NF-κB-associated genes are significantly downregulated in mixed glial cultures derived from mice with IKK KO microglia

We have previously reported a primary mixed glial culture derived from C57Bl/6 mouse pups, and have demonstrated that RML inoculum at low concentration (0.1%) is sufficient to induce infection and detect newly synthesized PrPSc, not from residual brain homogenate [27]. We used flow cytometry and western blot to characterize how mixed glia cultures differ between C57Bl/6 mice (referred to as wild-type, WT) and mice with IKK KO microglia. We performed flow cytometry analysis on two different batches of mixed glia derived from WT pups and those with IKK KO microglia, using GLAST as a marker for astrocytes and Iba1 as a marker for microglia. The percentage of Iba1 + microglia in the WT batches ranged from 15 to 35%, with an average of 25% [28]. The Iba1 + microglia from the batches with IKK KO microglia ranged from 0.59% to 0.94% (S1A and S1B Fig). Therefore, we will henceforth refer to mixed glia cultures derived from these animals as IKK KO microglia-limited cultures. These findings suggest that IKK in microglia is important for their survival and proliferation in culture [29,30]. Moreover, our cell culture model containing minimal microglia highlights their role in controlling prion replication *in vitro.*

To assess how our WT mixed glia and microglia-limited cultures respond to the milieu in the prion-infected brain, cultures were treated with 0.1% Normal Brain Homogenate (NBH) or Rocky Mountain Laboratories (RML) mouse-adapted scrapie brain homogenates for 7 days. Western blot analysis showed GFAP, an astrocyte marker, for both cell types, regardless of inoculum. However, Iba1 + microglia were only detectable by western blot in cultures treated with RML, and Iba1 expression was nearly undetectable in microglia-limited cultures (S1C Fig).

The IKK and NF-κB signaling pathways encompass a large number of genes. To focus on specific genes that were involved in microglial response to prion infection, we utilized a mouse NF-κB signaling pathway panel containing 84 genes of interest. WT and IKK KO microglia-limited cultures were treated for 7 days with NBH or RML-scrapie brain homogenates. Many NF-κB-associated genes are downregulated in IKK KO microglia-limited cultures compared to WT cultures, regardless of NBH- or RML-treatment (S2A Fig). A substantial difference in gene expression was observed between RML-infected WT and IKK KO microglia-limited cultures, while IKK KO microglia-limited cultures show a negative fold change of -2 or greater for 34 of the 82 genes analyzed (41%) (S1 Table). The greatest fold regulation was seen in toll-like receptor 9 (*Tlr9*) (-142) and *Bcl2a1a* (-146). Few genes increased, but the largest fold regulation increases were seen in epidermal growth factor receptor (*Egfr*) (+3.95) and macrophage colony-stimulating factor 1 (*Csf1*) (+3.25), involved in cell growth and microglia proliferation, respectively [31,32].

To best observe changes in gene expression between RML-infected WT and IKK KO microglia-limited cultures, analysis presented in a volcano plot highlights specific genes which show significance in pathways involved in prion disease (S2B Fig). Of note, many inflammatory cytokines and chemokines are downregulated in IKK KO microglia-limited cultures (*Tnfα*, *Il1α*, *Il1β*, *Ccl2* and *Ccl5*), as well as the anti-inflammatory cytokine *Il10*. Interestingly, the NLRP3-associated gene *Caspase-1* is significantly decreased. Of note, many TLRs, including *Tlr1*, *Tlr2*, *Tlr4*, *Tlr6* and *Tlr9*, are all decreased in IKK KO microglia-limited cultures. A full list of genes analyzed, including their fold change, is available in S1 Table.

**Fig 1:**
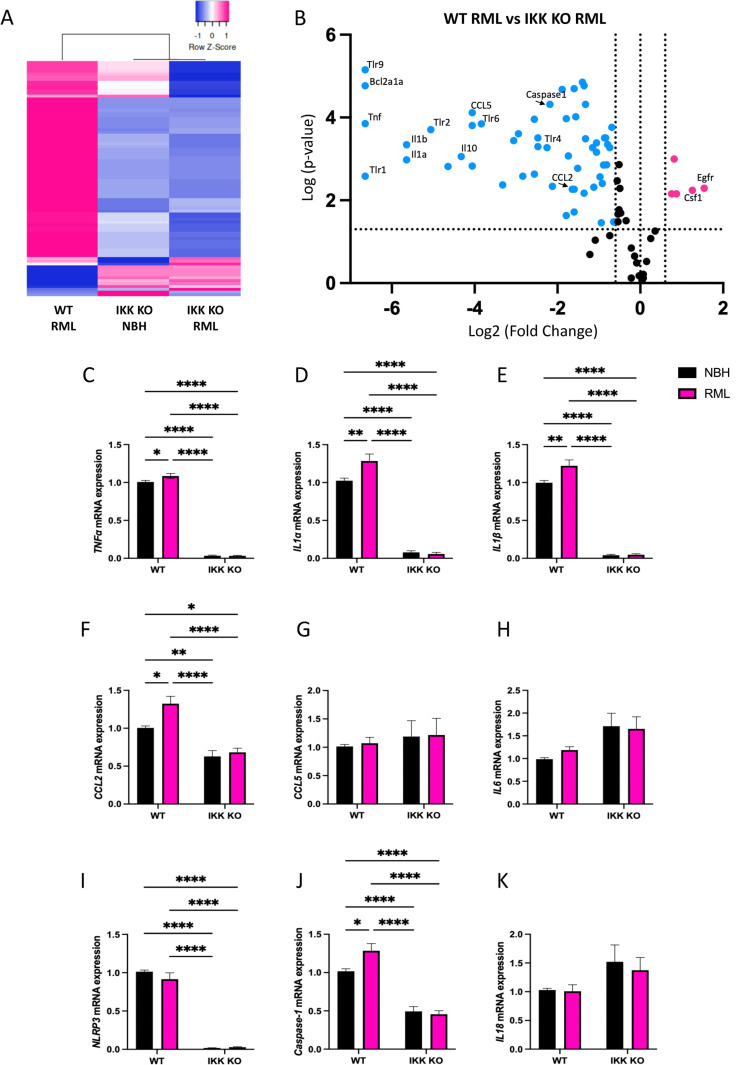
NF-κB-associated genes are significantly downregulated in mixed glial cultures derived from mice with IKK KO microglia. mRNA expression of the NF-κB-associated cytokines and chemokines **A** *TNFα*, **B** *IL1α*, **C** *IL1β*, **D** *CCL2*, **E** *CCL5* and **F** *IL6* were assessed. The NLRP3-associated genes **G** *NLRP3*, **H** *Caspase-1* and **I** *IL18* were assessed. Analysis of 3-5 biological replicates, each with 3 technical replicates. One-way ANOVA and post-hoc Tukey test, error bars = SEM, *p < 0.05, **p < 0.01, ****p < 0.0001.

Based on the findings of this panel, we focused on six NF-κB-related genes known to be upregulated in the prion-infected brain [8,9,33] to further validate the mRNA changes in glial cultures. Additionally, we included three NLRP3-associated genes to assess the downstream effects of the NF-κB pathway. A modest increase in NF-κB-associated genes were observed in RML-infected WT glia compared to NBH-treated WT glia, consistent with previous findings [27]. RML infection modestly but significantly upregulated *Tnfα*, *Il1β*, *Ccl2* and *Caspase-1* in WT mixed glia, but not IKK KO microglia-limited cultures. A significant decrease was identified for *Tnfα* (Fig 1A), *Il1α* (Fig 1B), *Il1β* (Fig 1C), and *Ccl2* (Fig 1D) in both NBH-treated and RML-infected IKK KO microglia-limited cultures compared to RML-infected WT cultures (*p* < 0.0001). No significant differences between WT and IKK KO microglia-limited cultures were observed for either NBH-treated or RML-infected groups for *Ccl5* (Fig 1E) or *Il6* (Fig 1F). A significant decrease was also seen in genes downstream of the NF-κB pathway, involved in the formation of the NLRP3 inflammasome. *Nlrp3* (Fig 1G) and *Caspase-*1 (Fig 1H) were both downregulated in IKK KO microglia-limited cultures compared to RML-infected WT cultures (*p* < 0.0001), but no significant differences were observed for *Il18* (Fig 1I).

Together, significant genes regulated by NF-κB, and influential in the downstream NLRP3 pathway, are upregulated in a primary mixed glial population when exposed to RML-infected brain homogenate. When the majority of microglia are removed from cultures, expression of many NF-κB-related genes decreases drastically, independent of exposure to prions.

### Mice with IKK KO microglia present accelerated prion disease

**Fig 2:**
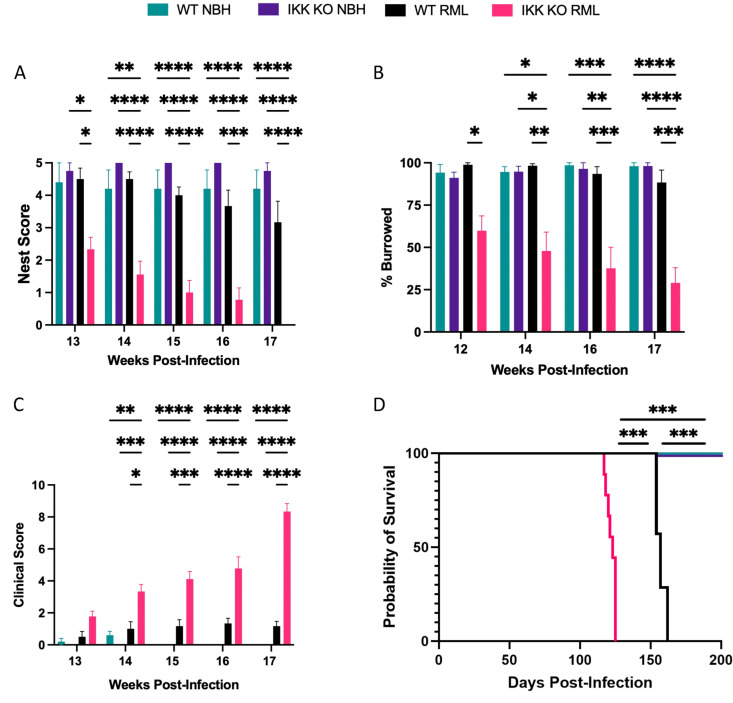
Mice with IKK KO microglia present accelerated prion disease. Mice were infected intracranially with RML mouse-adapted scrapie and monitored for changes in **A** nesting behavior, **B** burrowing behavior, and **C** clinical scores. **D** Animals displaying clinical scores of 10 were euthanized. NBH-treated groups contained 4 IKK KO and 5 WT mice, and RML-infected groups contained 9 IKK KO and 6 WT mice. For behavioral and clinical analyses, a two-way repeated measures ANOVA was used. For survival curve, a Log-rank (Mantel-Cox) test was performed. *p < 0.05, **p < 0.01, ***p < 0.001, ****p < 0.0001.

Degeneration of neurons in the hippocampus, particularly in the CA1 region, is a prominent sign of prion diseases such as RML mouse-adapted scrapie [34,35]. Mice intracranially inoculated (RML) were assessed for behaviors associated with hippocampal function – the ability to build nests and to burrow. Historically, wild-type (WT) mice do not show impaired nest building until 18 weeks post-infection (wpi) [36], whereas mice with IKK KO microglia built deficient nests in as little as 13 wpi (Fig 2A). Similar findings were seen in burrowing, as mice with IKK KO microglia decreased in burrowing behavior as early as 12 wpi (Fig 2B), while WT mice historically do not show a decline in burrowing until 17 or 18 wpi [36]. Although early clinical signs of prion disease began to manifest in WT mice at 13 or 14 wpi, we did not see significantly differences from NBH controls until 16 wpi, and signs did not warrant euthanasia (scoring a 10) until 23 or 24 wpi (Fig 2C) [36]. Mice with IKK KO microglia began showing significant clinical signs beginning at 13 wpi, and signs were advanced enough to perform euthanasia for all animals by 18 wpi (Fig 2C). No significant changes in weight were seen for any group as disease progressed (S3 Fig). All WT mice succumbed to disease by 23 wpi, with an average of 157 days post-infection (dpi) +/- 7 days, whereas mice with IKK KO microglia succumbed to disease by 18 wpi, with an average of 123 dpi + /- 8 days (*p* = 0.0002, Fig 2D).

### Removal of IKK in microglia induces change in microglia number and morphology during prion infection

**Fig 3:**
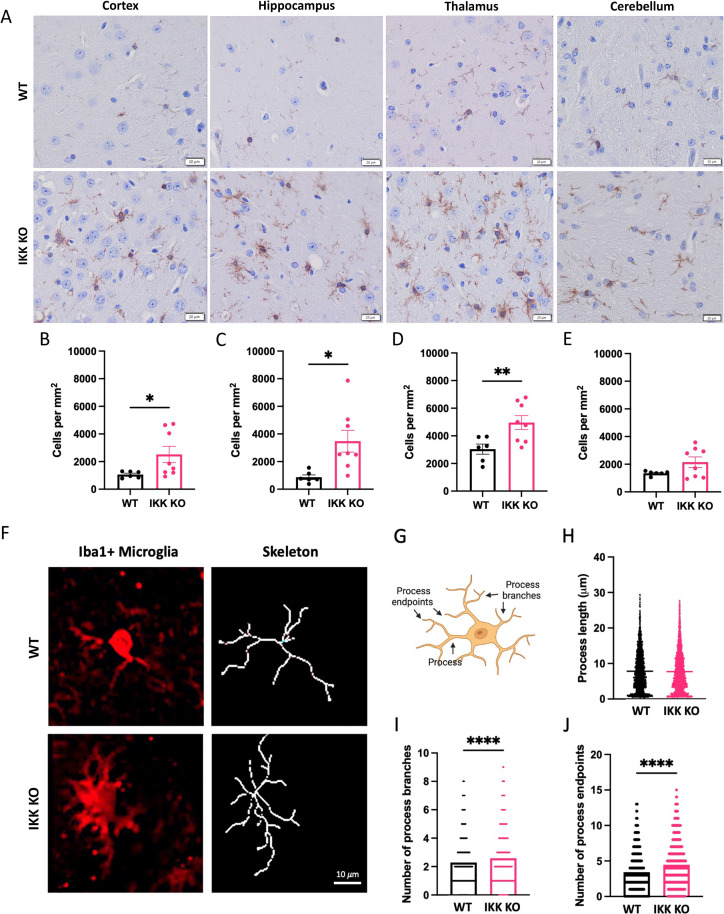
Removal of IKK in microglia induces changes in microglia number and morphology during prion infection. Terminal mice with IKK KO microglia were compared to wpi-matched WT infected mice. **A** Brains were stained for Iba1 + microglia, which were counted and compared in the **B** cortex, **C** hippocampus, **D** thalamus and **E** cerebellum for 9 animals with IKK KO microglia, 6 wpi-matched WT and 3 terminal WT animals. Scale bars = 20·m. **F** Representative images of Iba1 + hippocampal microglia morphological skeletons between groups. Scale bar = 10·m. **G** Microglia cartoon depicting features analyzed via skeletonization, created with Biorender.com. **H** Process length, **I** number of process branches and **J** number of process endpoints was compared between hippocampal microglia from WT mice and those with IKK KO microglia. Skeletonization and C3 analysis were performed on 3 randomly selected animals per group. Welch’s t-test, error bars = SEM, **p < 0.01, ****p < 0.0001.

Although generally considered to provide protection in the host, the role of microglia in prion pathogenesis remains poorly understood. It is well established that Iba1 + microglia increase in numbers, and that both microglia and astrocytes transition to an activated morphology over the course of prion infection [37–40] (S4 Fig). Mice with IKK KO microglia succumbed to prion infection around 17 wpi. To understand the pathological processes that contributed to this rapid disease onset in mice with IKK KO microglia, and determine whether their glial cells were displaying typical responses to prion infection, a cohort of prion-infected WT mice was euthanized at 17 wpi for comparison, referred to as wpi-matched WT mice. Additionally, comparisons were made between terminal mice with IKK KO microglia and terminal WT mice. Brain regions associated with significant prion deposition in RML scrapie were assessed for Iba1 + microglia in the frontal cortex, hippocampus, thalamus and cerebellum of prion-infected mice with IKK KO microglia and wpi-matched WT mice (Fig 3A) [37,41]. Significantly more Iba1 + microglia were detected in the hippocampus (*p* = 0.0070, Fig 3C), and thalamus (*p* = 0.0075, Fig 3D), but not the cortex or cerebellum (*p* = 0.0545, Fig 3B and *p* = 0.0719, Fig 3E, respectively), in mice with IKK KO microglia. Numbers of Iba1 + microglia in these animals was reminiscent of we and others have previously reported in WT mice at terminal disease [36,38], with no significant differences between the two strains with terminal prion disease (S5 Fig). Importantly, no significant differences were seen in Iba1 + microglia between WT mice and mice with IKK KO microglia when they were mock-infected with NBH (S6 Fig).

The morphology of Iba1 + hippocampal microglia was analyzed using skeletonization (Fig 3F) to assess process length, the number of process branches, and the number of process endpoints for each cell (Fig 3G) [36]. Although process length was not significantly different between groups (Fig 3H), the number of process branches (Fig 3I) and process endpoints (Fig 3J) were significantly greater in mice with IKK KO microglia compared to wpi-matched controls (*p* < 0.0001).

Together, these data show that mice with IKK KO microglia still have equivalent numbers of Iba1 + microglia in the brain. However, when intracranially infected with RML scrapie, these mice showed a rapid increase in the number of Iba1 + microglia in the cortex, hippocampus and thalamus compared to wpi-matched infected WT mice. Analysis of the morphology of hippocampal microglia showed more activation in IKK KO microglia compared to microglia from wpi-matched WT mice.

### Brains with microglial IKK KO show increased GFAP expression and activated astrocytes during prion infection

**Fig 4:**
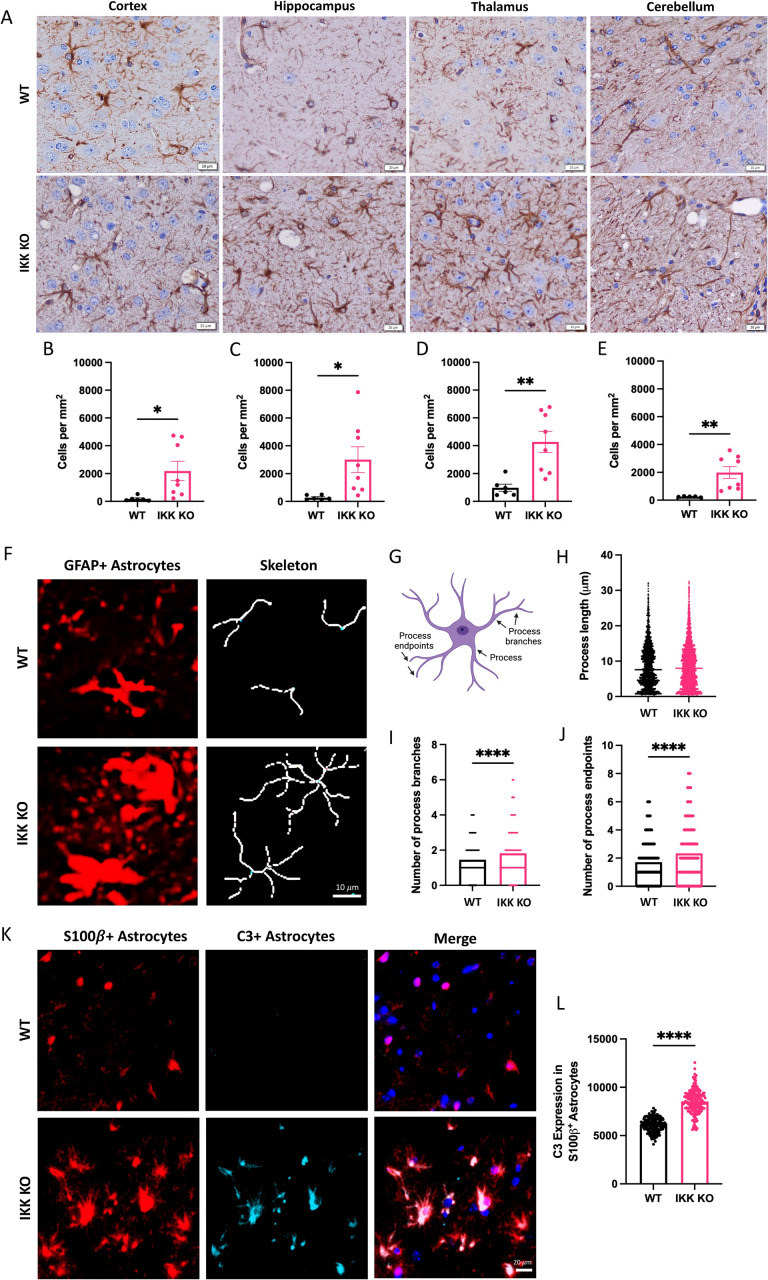
Brains with microglial IKK KO have increased GFAP expression and activated astrocytes during prion infection. Terminal mice with IKK KO microglia were compared to wpi-matched WT infected mice. **A** Brains were stained for GFAP+ astrocytes, which were counted and compared in the **B** cortex, **C** hippocampus, **D** thalamus and **E** cerebellum for 9 animals with IKK KO microglia, 6 wpi-matched WT and 3 terminal WT animals. Scale bars = 20·m. **F** Representative images of GFAP+ hippocampal astrocyte morphological skeletons between groups. Scale bar = 10·m. **G** Astrocyte cartoon depicting features analyzed via skeletonization, created with Biorender.com. **H** Process length, **I** number of process branches and **J** number of process endpoints was compared between hippocampal astrocytes from WT mice and those with IKK KO microglia. **K** Hippocampal astrocytes were co-stained for the pan-astrocytic marker S100β and the reactive astrocyte marker C3. Scale bar = 50·m. **L** Mean grey intensity of C3 was compared in S100β+ astrocytes (arbitrary units). Skeletonization and C3 analysis were performed on 3 randomly selected animals per group. Welch’s t-test, error bars = SEM, *p < 0.05, **p < 0.01, *** *p *< 0.001, ****p < 0.0001.

Both GFAP expression and C3 expression (a marker of reactive astrocytes) are known to increase throughout the brain over the course of prion infection [42–44]. GFAP+ astrocytes were counted in the frontal cortex, hippocampus, thalamus and cerebellum of prion-infected WT mice and mice with IKK KO microglia (Fig 4A). Compared to wpi-matched WT mice, significantly more GFAP+ astrocytes were detected in the cortex (*p* = 0.0020, Fig 4B), hippocampus (*p* = 0.0006, Fig 4C), thalamus (*p* = 0.0029, Fig 4D) and cerebellum (*p* = 0.0122, Fig 4E) in terminal mice with IKK KO microglia. These mice showed significantly more GFAP+ astrocytes in the hippocampus compared to terminal WT mice (*p* = 0.0147), but otherwise had similar GFAP+ astrocyte numbers to terminal WT mice (S7 Fig). No significant changes were seen in GFAP+ astrocyte numbers in mock-infected brains, except for the cortex where more cortical GFAP+ astrocytes were present in mice with IKK KO microglia compared to WT mice (*p* = 0.0027, S8 Fig).

The morphology of GFAP-expressing hippocampal astrocytes was analyzed using skeletonization (Fig 4F) to assess process length, the number of process branches, and the number of process endpoints for each cell (Fig 4G) [36]. Although process length was not significantly different between mice with IKK KO microglia and wpi-matched controls (Fig 4H), the number of process branches (Fig 4I) and process endpoints (Fig 4J) were significantly greater in mice with IKK KO microglia (*p* < 0.0001).

To assess the number of neurotoxic reactive astrocytes, present in the hippocampus [5,14,44], brains were stained for colocalization of the pan-astrocyte marker S100β and the complement cascade protein C3 (Fig 4K). Significantly more C3 was detected in S100β+ hippocampal astrocytes in mice with IKK KO microglia compared to wpi-matched WT mice (*p* < 0.0001, Fig 4L).

Healthy mice with IKK KO microglia have similar numbers of GFAP+ astrocytes in most brain regions analyzed. Infected mice with IKK KO microglia showed a rapid increase in the number of GFAP+ astrocytes in the cortex, hippocampus, thalamus and cerebellum compared to wpi-matched WT mice.

### Removal of IKK in microglia does not protect against prion-induced neuronal death *in vitro* or *in vivo*

**Fig 5:**
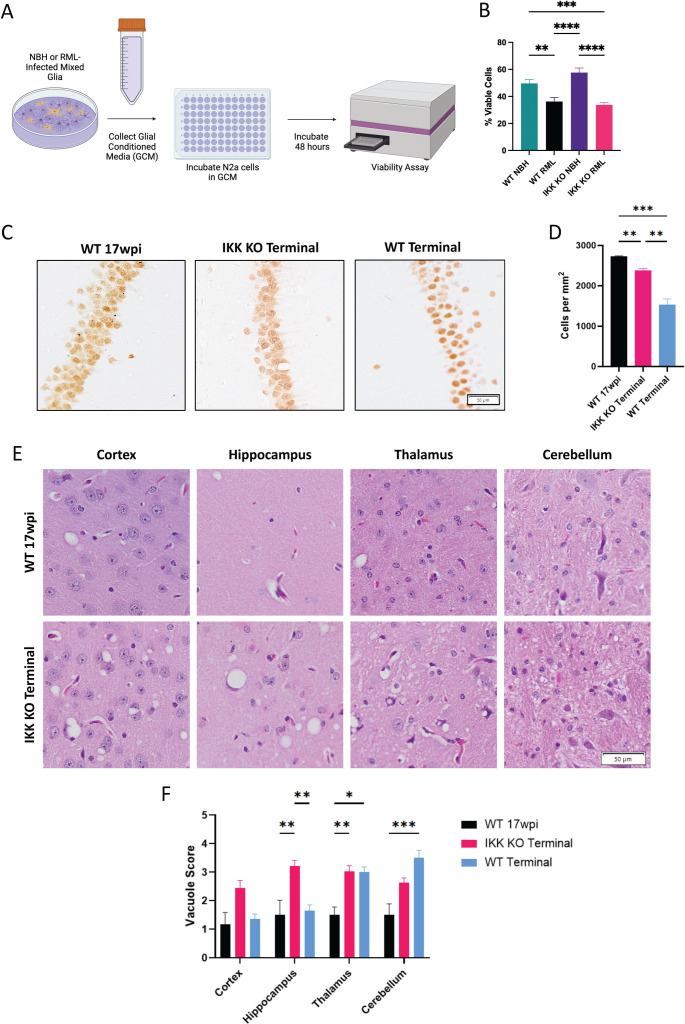
Removal of IKK in microglia does not protect against prion-induced neuronal death *in vitro* or *in vivo.* **A** Mixed glial cultures were treated for 7 days with NBH or RML homogenates. Glial conditioned media (GCM) was isolated and plated on N2as in 96-well plates and chamber slides for 48 hours. Image created with Biorender.com. **B** GCM-treated N2as were analyzed for percent viability using a Presto Blue cell viability assay. **C** Representative images of NeuN+ cells in the CA1 region of the hippocampus for terminal mice with IKK KO microglia (*n = *9), infected wpi-matched WT mice (*n = *6) and terminal WT mice (n = 7) and **D** CA1 NeuN+ neuronal counts. The severity of vacuolization for infected mice was scored from 1-5 by three blinded pathologists in the cortex, hippocampus, thalamus and cerebellum. **E** Representative images of vacuoles for terminal mice with IKK KO microglia and infected wpi-matched WT mice. **F** Bar graph showing mean vacuole severity for all infected mice. For the viability assay, a two-way ANOVA and post-hoc Tukey test was used. For NeuN counts, a Brown-Forsythe and Welch ANOVA was used. For vacuole counts, a two-way ANOVA and post-hoc Tukey test was used. Error bars = SEM, *p < 0.05, **p < 0.01, *** *p *< 0.001, ****p < 0.0001. Scale bars = 50·m.

To determine the effects of factors secreted into the media by WT mixed glia compared to IKK KO microglia-limited cultures, glial conditioned media (GCM) was harvested from NBH or RML-treated primary glial cultures after a 96-hour incubation. N2a neuroblastoma cells were incubated in GCM for 48 hours, then viability was measured with Presto Blue (Fig 5A). GCM from RML-infected WT cultures averaged 13.4% fewer viable cells (*p* < 0.01), and RML-infected IKK KO microglia-limited cultures averaged 23.9% fewer viable cells (*p* < 0.0001), compared to the NBH-treated samples (Fig 5B). However, there were no statistically significant differences in neuronal viability between RML-infected WT and IKK KO microglia-limited culture GCM.

Neuronal loss in prion-infected mice predominantly occurs in the CA1 region of the hippocampus [34]. To assess the number of living neurons, NeuN+ neurons were counted in the CA1 region to assess for neuronal loss. No differences were observed between the CA1 regions of hippocampi from mock-infected mice with WT and IKK KO microglia (S9A Fig). Significantly fewer NeuN+ neurons were detected in mice with IKK KO microglia, indicating significant hippocampal neuronal loss compared to infected wpi-matched WT mice (*p* = 0.0015, Fig 5C and 5D). Terminal WT mice had significantly fewer NeuN+ neurons compared to both terminal mice with IKK KO microglia and infected wpi-matched WT mice (*p =* 0.0014 and *p* = 0.0004, respectively).

A pathological hallmark of prion disease is the development of a spongiform pattern within the brain, referred to as vacuoles [45,46]. Brains were stained with hematoxylin and eosin (H&E) to assess for severity of vacuoles based on both size and number in the frontal cortex, hippocampus, thalamus and cerebellum (Fig 5E). Vacuole severity was significantly higher in the hippocampus of infected mice with IKK KO microglia compared to infected wpi-matched and terminal WT mice (*p = *0.0015 and *p* = 0.0029, res*p*ectively), and higher in the thalamus compared to wpi-matched WT mice (*p = *0.0079). Vacuole severity was higher in the cerebellum of terminal WT mice compared to 17wpi WT mice (*p = *0.0003) (Fig 5F). No significant vacuoles were seen in any of the above brain regions for mock-infected mice (S9B–S9E Fig).

Infected mice with IKK KO microglia presented with signs of neurodegeneration and vacuolization at an accelerated pace compared to wpi-matched WT mice. The cell viability assay shows that cells from both WT mixed glia and IKK KO microglia-limited cultures are secreting neurotoxic factors, even when very few microglia are present.

### IKK KO in microglia leads to increased accumulation of PK-resistant PrP *in vitro* but decreased accumulation *in vivo*

**Fig 6:**
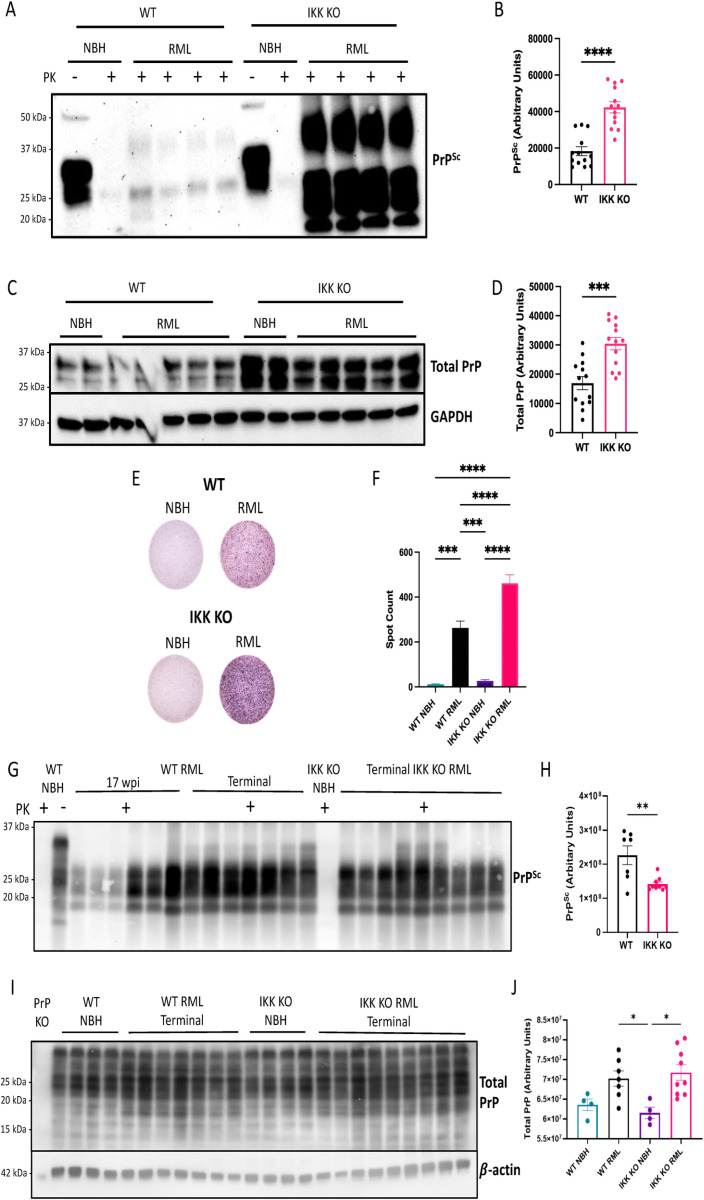
IKK KO in microglia leads to increased accumulation of PK-resistant PrP *in vitro* but decreased accumulation *in vivo.* Mixed glial cells containing WT astrocytes and WT or IKK KO microglia were treated with NBH or infected with RML for 7 days. **A** Cells were lysed and digested with PK for PrP^Sc^ observation, undigested (Total) PrP and GAPDH as a control. **B** protease-resistant protein signal for PrP^Sc^ and **C** protein signal for total PrP was measured. **D** Infected cells were trypsinized and transferred to an ELISpot plate for a scrapie cell assay to **E** count the number of infected cells (normalized). Prion western blot analysis and spot counts are combined data from three separate experiments. **F** Homogenized brains from terminally infected mice with IKK KO microglia and both wpi-matched and terminally infected WT mice were digested with PK and **G** protease-resistant protein signal was measured using Sha31 antibody comparing terminal groups. **H** Total PrP was assessed using Sha31 antibody for both terminally infected and mock-infected mice and **I** compared between groups. For western blot quantification of PrP^Sc^, an unpaired t-test was performed. For scrapie cell assay, a two-way ANOVA and post-hoc Tukey test was used. For western blot analysis of total PrP, a one-way ANOVA and post-hoc Tukey test was used. Error bars = SEM, *p < 0.05, **p < 0.01, *** *p *< 0.001, ****p < 0.0001.

Cell lysates from infected primary mixed glia containing WT mixed glia and or IKK KO microglia-limited glia were analyzed for infectious, PK-resistant PrP (denoted PrP^Sc^) or total PrP (containing both PrP^C^ and PrP^Sc^) via western blot (Fig 6A). Comparison of band densitometry revealed a significant increase in PrP^Sc^ (*p* < 0.0001, Fig 6B) and total PrP (*p* < 0.001, Fig 6C) from lysates with limited microglia compared to WT mixed glia.

Infected glial cultures were quantified for prion plaques using a scrapie cell assay (Fig 6D). Significantly more plaques (spots) were detected in RML-infected WT cells compared to the NBH-treated WT cells (*p* < 0.001) and in RML-infected IKK KO microglia-limited cells compared to NBH-treated IKK KO microglia-limited cells (*p* < 0.0001). Similar to the western blot analyses, there were significantly more prion plaques present in RML-infected IKK KO microglia-limited cultures compared to WT mixed glial cultures (*p* < 0.0001) (Fig 6E).

To determine if this phenomenon was retained *in vivo*, terminal animals with IKK KO microglia and both wpi-matched and terminal WT animals were analyzed for prion accumulation in the brain. The left hemisphere was homogenized and assessed for PK-resistant PrP by immunoblot. Multiple antibodies were used to probe for PrP, including an N-terminal antibody (12B2) and a C-terminal antibody (Sha31) (antibody epitopes shown in S10A Fig). Interestingly, no differences were seen in band densitometry or glycosylation pattern between infected terminal mice with IKK KO microglia and wpi-matched WT brains using either 12B2 (S10B and S10C Fig) or Sha31 (S11A and S11B Fig). No changes were seen in total PrP in these brains with Sha31 (S11C Fig). At this time point there were no detectable differences in total PrP between NBH control and RML infected WT brains, whereas there was significantly more total PrP in RML-infected brains with IKK KO microglia compared to their respective NBH controls (*p* < 0.001, S11D Fig).

The C-terminal antibody, Sha31, was used to analyze prion accumulation comparing terminal WT mice and terminal mice with IKK KO microglia. Significantly more PK-resistant PrP was detected in terminal WT mice compared to terminal mice with IKK KO microglia (*p* < 0.0045, Fig 6F and 6G). Band densitometry showed amounts of total PrP to be the same between terminal WT and mice with IKK KO microglia (Fig 6H and 6I). Assuredly, brains from mice with IKK KO microglia showed a decrease in IKKβ of approximately 50% compared to WT brains at terminal stages, which was independent of infection (S12 Fig).

## Discussion

Exact neuropathological mechanisms of prion disease remain incompletely understood. Expression of PrP is required for infection and critical for neuronal loss [40,47–49], but glial cells also play a distinct role in disease pathogenesis [43,50,51]. Neurons and astrocytes express the highest amount of PrP in the body and brain [52], and therefore are easily infected with PrP^Sc^ and can traffic it between cells [53]. Microglia scavenge and phagocytose PrP^Sc^, which results in a phenotypic change from scavenging and ramified to activated and amoeboid, often denoted as a shift from M2 to M1, or homeostatic microglia to disease-associated microglia (DAM) [6,38,40,41,50,54]. This induces the secretion of factors such as pro-inflammatory cytokines and chemokines, which in turn can induce astrocytes to become reactive and produce inflammatory and neurotoxic mediators [5,14,33,55].

Animal models of prion disease which have a reduction or elimination of microglia have varying effects on disease outcome, depending on the time point in which the reduction occurs. Generally, microglia are found to be protective to the host, and disease worsens when microglia are decreased or removed [25,39,41,56–58]. However, genetic manipulation to change the inflammatory state of astrocytes did not successfully extend the lives of mouse models [5,58]. Together, these findings suggest that inflammation from glial cells plays a critical role in host protection but can become detrimental if left unchecked. Therefore, reduction, but not elimination, of glial-induced inflammation is a promising avenue for therapeutics [27,59–61]. Developing effective treatments requires elucidating specific inflammatory signaling pathways from astrocytes and microglia that affect neuronal health in the prion-infected brain.

Microglia, potent regulators of prion infection, are involved in host protection through inflammatory signaling, communication with astrocytes, phagocytosis and degradation of PrP^Sc^ [39,40,56–58,62]. Many genes involved in the NF-κB signaling pathway are highly upregulated in animal models with prion disease [4,8,9], suggesting that this innate immune pathway responds to prion infection. To date, no studies have analyzed the role of microglia-specific NF-κB signaling in prion-infected mice. One study removed IKK from cells in the CNS with neuroectodermal lineage – namely neurons, oligodendrocytes, and astrocytes. No changes in disease time-course, PrP^Sc^ accumulation or astrogliosis were found and the authors concluded that NF-κB does not play a significant role in prion-induced inflammatory signaling [13]. Another study directly knocked out the subunits of NF-κB in cells derived from the neuroectoderm. This group saw a decrease in survival in these mice upon prion inoculation, and increased apoptosis [12]. Both studies failed to account for microglia, which are derived from the mesoderm [63] and known to be critical regulators of NF-κB-associated inflammation in prion and other diseases [5,14,33,58]. Here, we utilize both a primary glia culture model, as well as a mouse model with IKK KO microglia, to characterize the role of microglia-specific IKK and NF-κB signaling in prion disease.

Cx3Cr1Cre mice that express Cre recombinase under the *Cx3cr1* promoter in the mononuclear phagocyte system and were combined with floxed IKKβ mice [26] to generate mice with IKK knockout (KO) specific to mononuclear phagocytes (macrophages, monocytes and microglia). As macrophages and monocytes are rarely found in the brain in prion disease [33], these mice are effectively microglia-specific IKK KO. In an innate immune response, IKK is critical for NF-κB translocation to the nucleus for transcription [19,20], making these cells ineffective at NF-κB signaling. However, importantly, all other brain cells in these mice have functional IKK and NF-κB signaling. Analysis of brain homogenates by western blot reveal a 50% decrease in IKKβ in mice with IKK KO microglia compared to WT brains (S12 Fig).

Even a small number of microglia can greatly impact a cell culture system, especially in modulation of astrocytes [64]. There is significant signaling crosstalk between astrocytes and microglia, as astrocytes remain relatively unresponsive to environment changes in the absence of microglia-derived signaling [14,17], and microglia rely heavily on astrocytes to promote their proliferation [31]. Previously, we reported a mixed glial culture model from C57Bl/6 (WT) mouse pups can become infected by mouse-adapted scrapie [27]. Although there is some batch-to-batch variation, these cells are composed predominantly of astrocytes and contain approximately 25% microglia, which is similar to what has been reported previously [28]. When mixed glial cultures were derived from the microglia-specific IKK KO mice, the number of microglia present in the cultures dropped to less than 1% (S1 Fig). We suspect this may be due to the role of IKK and NF-κB signaling in cell proliferation and survival [30]. Indeed, *Bcl2a1a,* which has a potent anti-apoptotic role in cells, was one of the most downregulated genes observed in the cultures with IKK KO microglia [65]. Although the lack of sufficient microglia in these cultures prevents us from being able to parse out the specific role of IKK in microglia, it does allow for comparisons between a culture model with healthy microglia and one with few microglia with limited NF-κB signaling abilities. We infected the WT and IKK KO microglia-limited cultures with RML-scrapie prion brain homogenates to assess their NF-κB signaling, neurotoxic effects, and PrP^Sc^ accumulation.

Approximately 41% of the NF-κB-associated genes analyzed in the primary mixed glia cultures were downregulated (fold regulation of -2 or less) in RML-scrapie-infected IKK KO microglia-limited cultures compared to RML-infected WT mixed glia (S2 Fig and S1 Table for complete list). This included many toll-like receptors (TLRs), particularly *Tlr1*, *Tlr2*, *Tlr4*, *Tlr6* and *Tlr9*, all of which are highly expressed in microglia. Decrease in TLR expression is consistent with a decrease in microglial numbers, and also likely contributes to an overall decrease in inflammatory molecules [66], as signaling is dysregulated in the absence of sufficient TLRs. *Tlr3* is not significantly downregulated, but this TLR is highly expressed in astrocytes, along with low-level expression of *Tlr1*, *Tlr4*, *Tlr5* and *Tlr9* [67]. Interestingly, microglia are critical for TLR4 signaling in astrocytes, and important for optimal signaling through TLR2 and TLR3 [68], which may contribute to overall downregulation of astrocyte-specific signaling in cultures containing limited IKK KO microglia, as these microglia cannot fully prime the astrocytes. TLR4 is one of the best studied TLRs, as its stimulation by signals such as LPS induce NF-κB inflammation in microglia [20,66], which is critical to elicit an astrocyte response [68]. In prion mouse models, *Tlr1*–*9* are all upregulated in the brain at terminal stages of disease [66]. TLR2 and TLR4 signaling are important for sensing damage-associated molecular patterns (DAMPs) and has been shown to be implicated in prion disease, as knocking out either of these receptors in mice accelerates disease [69,70].

Of the other downregulated genes, many were inflammatory cytokines and chemokines known to be upregulated in the prion-infected brain [8,9,33]. From this panel, we focused our attention on a select number of NF-κB-related genes known to be involved in prion disease, and downstream NLRP3-related genes [8–11,71]. We performed qPCR analysis on three to five sets of RML-infected primary mixed glia, each isolated from a separate set of mouse pups (Fig 1). The cytokines Tnfα, Il1α and Il1β, are predominantly produced by microglia and influence the state of astrocyte reactivity [5,14]. *Tnfα*, *Il1α* and *Il1β* were consistently downregulated in IKK KO microglia-limited cultures, as were the NLRP3-associated genes *Nlrp3* and *Caspase-1*, suggesting that these genes are activated predominantly by NF-κB within microglia. CCL2 is produced by astrocytes and promotes an M1 phenotype in microglia [72]. We saw an overall downregulation in *Ccl2*, although less robust than the other genes. As this chemokine is produced mainly by astrocytes [72,73], it is likely still expressed in the absence of sufficient microglia in the IKK KO cultures. This suggests that in the absence of sufficient microglia and microglia-specific NF-κB signaling, signaling from astrocytes is also decreased, as microglia are critical for astrocytes to reach a full activation state [74]. These results highlight that crosstalk and reciprocal activation of microglia and astrocytes are important for proper function of both cell types in response to infection.

Due to known involvement in NF-κB signaling and prion pathogenesis, we additionally looked at expression of *Ccl5* and *Il6*, as well as the NLRP3-associated gene, *Il18*. A large variation in expression of these genes with each set of glia analyzed showed both significant downregulation and, intriguingly, significant upregulation, in IKK KO microglia-limited cultures compared to WT mixed glial cultures, suggesting compensatory signaling pathways from astrocytes that contribute to neuroinflammatory response. However, unlike many of the other genes, no significant changes were seen between the WT glia treated with normal brain homogenate (NBH) or RML, suggesting that this response is not directly induced by prions.

We demonstrate that cultures with a limited number of microglia have drastically different responses to treatment with prions. They have decreased markers of NF-κB and NLRP3 signaling (Fig 1), but are equally neurotoxic to cultures containing microglia (Fig 5) and have significantly more prion accumulation (Fig 6). Together, these findings support previous studies demonstrating a protective role for microglia in prion infection [25,39,57,58]. However, due to the immature brains these cells are derived from, and the limited number of microglia in these primary cultures, this model is not robust enough to address the specific role of microglial IKK and NF-κB signaling in an adult animal in prion disease. To better interrogate this, we turned to an *in vivo* model.

Conversely to our primary cell culture model, healthy mice with IKK KO microglia show comparable numbers of microglia as WT mice (S6 Fig). Despite KO microglia lacking IKK2 and therefore having non-functional canonical NF-κB signaling, the Iba1 + microglia in prion-infected mice with IKK KO microglia increase in numbers more rapidly than those in wpi-matched infected WT mice. Significantly more Iba1 + microglia were observed in most of the analyzed brain regions, most of which were characteristic of M1 microglia or DAM, having a large amoeboid shape with increased processes and process branches, whereas those from WT mice still resembled an M2 phenotype, with fewer processes and a smaller cell body (Fig 3). This suggests that microglia are still reacting to the prion-infected brain even in the absence of IKK and NF-κB signaling, and that their activation state may be dysregulated. Further interrogation is required to determine if this activated phenotype is a direct or indirect response to IKK knock-out.

Accumulation of PrP^Sc^, microgliosis and development of vacuoles have been shown to have a strong temporal correlation in WT mice infected with RML-scrapie, particularly in the hippocampus and thalamus, with the strongest pathology beginning around 125 days post-infection (approximately 18 wpi) [37]. This correlates with what we previously observed for the onset of hippocampal-related behavioral deficits and clinical signs [36]. We observed that RML-infected mice with IKK KO microglia began to show behavioral and clinical signs of disease as early as 12 wpi, and reached terminal stages of disease before WT mice began to show significant signs of disease (Fig 2). Further analysis is required to determine the preclinical stages of prion disease for mice with IKK KO microglia to assess if their gliosis, prion accumulation, spongiosis and neuronal loss are similar to WT mice.

GFAP expression increased in all brain regions analyzed in terminally infected mice with IKK KO microglia compared to wpi-matched infected WT mice. RML-infected mice with IKK KO microglia had more reactive astrocytes in the hippocampus, demonstrated by increased C3 expression and morphology [5,14](Fig 4). This suggests that NF-κB signaling from microglia may be a critical regulator of astrocyte activation states in prion pathogenesis, and astrocytes may be compensating in response to dysfunctional microglia [58].

Studies demonstrate that decreasing or fully ablating microglia in prion-infected animals leads to accelerated disease, increased vacuolization and astrogliosis [39,58]. Astrocytes from microglia-ablated mice showed enhanced ability to phagocytose neuronal synapses and increased unfolded protein response, both of which contribute to irreversible neuronal loss [35,58]. TNFα and IL1α are decreased in prion-infected mice without microglia, suggesting that microglia are a key source of NF-κB-related proinflammatory cytokines [39]. These microglia-derived cytokines, alongside C1qa, are responsible for inducing astrocyte reactivity, marked by increased C3 expression [14]. Increased inflammatory signaling by microglia is consistent with its role as the main antigen presenting cell with associated helper cell-like functions in the brain. Knockout of TNFα, IL1α and C1qa in prion-infected mice, although sufficient in ablating C3 + astrocytes, also decreased survival time and the number of homeostatic microglia [5]. Together, these studies show that microglia and astrocytes tightly regulate one another in the prion-infected brain and unbalancing this relationship leads to dysfunction and accelerated disease.

Microglial-induced inflammation is often cited as an inducer of neuronal cell death, either directly or indirectly [5,14,17,54,58,75,76]. Therefore, it was surprising that glial conditioned media (GCM) from infected WT and IKK KO microglia-limited glial cultures showed similar neurotoxicity to N2a cells in cell viability assays (Fig 5A and 5B). This suggests that astrocytes are secreting neurotoxic factors independently of microglia. These findings were consistent with our *in vivo* data, which showed neuronal death both directly and indirectly in animals with IKK KO microglia. Significantly fewer NeuN+ neurons were observed in the CA1 region of the hippocampi in terminally infected mice with IKK KO microglia compared to wpi-matched infected WT mice. This was consistent with the decrease in burrowing and nesting behavior, both behaviors that are related to neuronal health in the hippocampus. Terminal WT mice had significantly fewer NeuN+ neurons compared to terminal mice with IKK KO microglia (Fig 5C and 5D). There is evidence that vacuole formation is an indirect marker of neuronal death [46,77], and terminally infected mice with IKK KO microglia showed significantly more vacuolization in the hippocampus than either wpi-matched or terminally infected WT mice (Fig 5E–5I). This finding is consistent with a recent study that described increased vacuoles but no changes in CA1 hippocampal neurons in prion-infected mice lacking microglia [58]. Together, our findings suggest that IKK and NF-κB signaling from hippocampal microglia are critical for regulating astrocytes and protecting hippocampal neurons [58]. Further interrogation is required to understand this complex cellular crosstalk between these cell types.

Cellular responses to misfolded proteins are impaired in the primary cell cultures containing limited microglia, demonstrated by increased accumulation of PrP^Sc^ via western blots and scrapie cell assays (Fig 6A–6E). Intriguingly, this phenomenon is not observed when looking at crude brain homogenates from infected mice. Brains from terminally infected mice with IKK KO microglia show no difference in either PK-resistant PrP or total PrP compared to wpi-matched infected WT mice (S11 Fig), but decreased amounts of PK-resistant PrP and total PrP compared to terminal WT mice (Fig 6). This suggests that mice with IKK KO microglia succumbed to disease quicker than their brains accumulate misfolded prions, suggesting that neuronal death is induced by factors besides PrP^Sc^, such as abnormalities in glia. This phenomenon has been described for other mouse models. Mice with total microglia ablation succumb to disease due to astrocyte dysfunction, despite having less PrP^Sc^ compared to controls [58].

Our glial culture system suggests an integral role for microglia in PrP^Sc^ clearance. However, we are not seeing this obviously recapitulated *in vivo*. The prion-infected brain contains a variety of different types and activation states of microglia and astrocytes [40], which may be absent in our culture system. The presence of neurons, particularly those infected by PrP^Sc^, may shift the response of astrocytes and microglia [53,78]. Further investigation is necessary to understand what compensatory mechanisms are in the brain that may be regulating PrP^Sc^ clearance.

Accumulation of misfolded proteins is commonly associated with dysregulation of autophagy and lysosomal function. Rescue of either of these pathways is shown to improve life expectancy and clinical pathology in prion-infected mice [79–81]. Misfolded proteins are first identified and ubiquitinated by chaperone proteins, leading to the binding and sequestration of aggregates by p62, a protein upregulated by the NF-κB pathway [82,83]. Moreover, signals that activate the IKK complex are shown to initiate autophagy even in the absence of downstream NF-κB signaling, suggesting that knockout of IKK leads to impaired autophagy regardless of NF-κB-induced p62 signaling [84]. Increased abnormal protein accumulation was demonstrated in mice with IKK KO microglia in a model of rotenone-induced Parkinson’s disease (PD). However, conversely to our findings, there was a significant reduction in reactive astrocytes and neuronal loss in rotenone-treated male mice with microglial IKK KO, as well as a decrease in clinical signs of PD [3]. Studies have demonstrated that microglia are the predominant source of NF-κB signaling in animal models of neurodegeneration such as amyotrophic lateral sclerosis and tauopathy, and inhibition of this pathway in microglia in these models proves neuroprotective [1,2]. Knocking out IKK2/IKKβ in microglia in a mouse model of tauopathy led to increased accumulation of tau in microglia, but prevented its seeding and further spreading, ultimately leading to neuroprotection [1]. These studies indicate IKK as a critical mediator of aggregated protein modification and clearance. Our findings show a drastic increase in PrP^Sc^ in glial cultures with limited microglia compared to mixed glial cultures. Conversely, mice with IKK KO microglia showed decreased levels of total brain PrP^Sc^. Further studies are underway to tease out the mechanism by which IKK and microglia may be involved in prion accumulation and clearance.

Further characterization of this model is necessary to understand how microglia-specific IKK KO affects NF-κB signaling, as well as downstream effects such as the involvement of other inflammatory pathways, autophagy, and neuronal cell death. Although we see profound changes in our cell culture and mouse model in response to prion infection, it is difficult to tease out whether this is due to the role microglial IKK plays directly on protein clearance in glial cells, the lack of sufficient NF-κB inflammatory signaling, the lack of robust microglial numbers, or a combination of the three. More intriguingly, we do not see increased accumulation of PK-resistant prions in the brains of infected mice with IKK KO microglia. These mice show similar amounts of PrP^Sc^ compared to wpi-matched WT mice, and less PrP^Sc^ compared to terminal WT mice. However, neurons are rapidly deteriorating, and infection is fatal in mice with IKK KO microglia at a much earlier timepoint suggesting that there are other factors contributing to neurotoxicity other than prion accumulation – likely due to dysfunctional microglia and their crosstalk with astrocytes. Future studies will focus on disease progression by analyzing glial and neural morphology and function, prion propagation and clearance in prion-infected mice with IKK KO microglia prior to their display of clinical signs. This model gives insight to an age-old question – is it prion aggregation and accumulation, or downstream effects such as inflammation and gliosis, that leads to neurodegeneration? Understanding the involvement of IKK and NF-κB signaling in microglia may uncover both biomarkers and therapeutic targets for prion diseases.

## Methods

### Ethics statement

Cx3Cr1Cre-IKKflox mice (C57Bl6/J background, Cx3CrlCre available from Jax #025524) were kindly provided to us by Dr. Ronald Tjalkens [3]. All mice were bred and maintained at Lab Animal Resources, accredited by the Association for Assessment and Accreditation of Lab Animal Care International. All protocols are in approved by the Institutional Animal Care and Use Committee at Colorado State University. An approximately equal number of male and female mice were used throughout the study.

### Isolation of mixed glia

Zero to two-day old C57Bl6/J or CX3CR1-IKKflox pups were euthanized, and brains were extracted. Cerebellum, midbrain and hippocampus were removed and discarded, and the brains were placed in MEM/EBSS (Hyclone) containing 2x penicillin/streptomycin/neomycin (PSN) (Sigma) on ice. Cortical tissue was dissociated using 1.5 U/ml Dispase to isolate mixed glial cultures, as described previously [27]. 10^6^ mixed glial cells were plated in 10 cm dishes and cells were incubated at 37 º C with 5% CO_2_ in glial growth media (MEM/EBSS + 10% FBS + 1% PSN). Media was replaced after 24 hours and changed weekly.

### Flow cytometry

Confluent glial cultures were harvested at passage 1 with Cellstripper (Corning) at 37C for 15 mins. Cells were scraped and pooled into a conical tube and spun at 4°C for 5 mins at 400x*g*. Cells were rinsed with FACS buffer (sterile PBS with 0.5% BSA), spun again and counted on a hemocytometer. Cells were resuspended in FACS buffer at 1x10^6 cells/ml for staining. 100 μl was separated out for each reference control (unstained, PE + , PE-, FITC + , FITC-, live, and dead), and remaining cells received both GLAST-PE (Miltenyi Biotec, 1:400 dilution) and CD11b-FITC (BD Biosciences, 1:50 dilution). Cells were incubated with antibodies in the dark at room temperature for 1 hour. “Dead” cell control was placed on the heat block at 65C for 30 minutes, then after returning to room temperature, these were mixed with “live” unstained cells. Tubes containing cells were spun down and washed 3 times with FACS buffer. Cells were resuspended in 500 μl FACS buffer and transferred to library tubes on ice. SYTOX AADvance Dead Cell Stain (Invitrogen) (1:100 dilution) was added to Live/Dead reference control and all samples. The Cytek 4-laser Aurora Cytometer was used and settings were adjusted based on unstained cells and reference controls. Single cells were selected using FS Height x FS Area, then 10,000 cells were analyzed per reference control before performing live unmixing. For each sample, 50,000 live, single cells were analyzed. Data was processed in FlowJo, gating on single cells, live cells, then FITC-positive and PE-positive cells.

### Infection of primary glial cell cultures

For *in vitro* prion infection, mixed glia were plated at 10^5^ cells per well in 6-well plates and infected at 80% confluency with 0.1% normal or RML brain homogenate in media. Media was removed 72 hours later and cells were washed twice with PBS prior to fresh media being added to remove any residual brain homogenate. Supernatants (glial conditioned media (GCM)) and protein or RNA were extracted from plates after an additional 96 hours (7 days from initial homogenate treatment) and analyzed as described below.

### Reverse transcriptase quantitative PCR analysis

RNA was extracted from 6-well dishes using cell scraping, QIAshredder and RNeasy extraction kits, in accordance with manufacturer’s protocol, including a DNase digestion step with the RNase free DNase kit (Qiagen, Valencia, CA). Purity and concentration were determined using a ND-1000 spectrophotometer (NanoDrop Technologies, Wilmington, DE). Following isolation and purification, 25 ng per sample of RNA was reverse transcribed using the iScript Reverse Transcriptase kit (BioRad, Hercules CA). cDNA was amplified within 24 hours of reverse transcription using iQ SYBR Green Supermix (BioRad, Hercules CA). For NF-κB panel, the RT^2^ profiler PCR array for Mouse NF-κB Signaling Pathway (Qiagen) was used with Iq Sybr (Bio-Rad), following manufacturer’s protocol. Each treatment was done in triplicate. Plates were analyzed using the Bio-Rad CFX96 Real-Time System. Files were uploaded into RT^2^ profiler PCR array GeneGlobe analysis software for quality control and statistical analysis. Using this software, samples were normalized to 3 housekeeping genes– *Hsp90ab1*, *Gapdh* and *β-actin*. This panel evaluated multiple replicates within a single batch of glia. To analyze specific genes across multiple cell batches, corresponding validated primer sequences were used for each gene at 10mM. Plates were analyzed using the LightCycler 480 II (Roche). The expression data was analyzed using the 2-ΔΔCT method and normalized to expression of reference genes *β-actin.* The fold difference was compared to control (WT normal brain homogenate treated) samples [85]. All treatments were done in triplicate samples and cDNA from each sample was measured in triplicate. Data is a combination of three to five biological replicates (separate batches of glia). Validated primer sequences are listed in Table 1.

**Table 1:** Primer sequences for reverse transcriptase quantitative PCR.

Gene	Forward primer	Reverse primer
TNFα	CCGATGGGTTGTACCTTGTC	AGATAGCAAATCGGCTGACG
IL1α	AGACGGCTGAGTTTCAGTGAG	TCTGGGTTGGATGGTCTCTTC
IL1β	GCAGCAGCACATCAACAAG	CACGGGAAAGACACAGGTAG
CCL2	TTAAAAACCTGGATCGGAACCAA	GCATTAGCTTCAGATTTACGGGT
CCL5	TTAAAAACCTGGATCGGAACCAA	TCGAGTGACAAACACGACTGC
IL-6	CTGCAAGAGACTTCCATCCAG	AGTGGTATAGACAGGTCTGTTGG
NLRP3	CCTGGGGGACTTTGGAATCA	GACAACACGCGGATGTGAGA
Caspase-1	AACCACTCGTACACGTCTTGC	ATCCTCCAGCAGCAACTTCA
IL18	GACTCTTGCGTCAACTTCAAGG	GTTGTCTGATTCCAGGTCTCCA
β-actin	GCTGTGCTATGTTGCTCTAG	CGCTCGTTGCCAATAGTG

### Cell viability assay

At 7 days post-infection, glial conditioned media (GCM) was removed from mixed glia, centrifuged at 1000 x g for 5 minutes at 4° C to pellet cell debris, and transferred to a fresh tube that was stored at -80° C for viability assays. GCM was thawed in a water bath at 37° C prior to use. N2a neuroblastoma cells were plated on black 96 well Nunc plates (Thermo Fisher Scientific) at 20,000 cells per well. 24 hours after plating, media was removed from N2a cells and replaced with GCM (8–12 replicate wells per treatment group). For each plate, fresh glial media was used as a control for live cells, and glial media containing 0.1% ethanol was used as a control to achieve dead cells. N2as were incubated with GCM or control media for 48 hours. PrestoBlue Cell Viability Reagent (Thermo Fisher Scientific) was allowed to reach room temperature and diluted 1:10 with fresh glial media. Cells were washed gently with PBS and 50 ·l PrestoBlue in media was added per well and incubated for 10 minutes at 37° C. Cells were analyzed using the FLUOstar Omega Plate Reader (BMG Labtech). Data was normalized to the live cell control.

### Scrapie cell assay

Primary mixed glial cells were infected with 0.1% RML or normal brain homogenate (NBH), as described above. 7 days later, cells were trypsinized and 50,000 cells were transferred to an ELISpot plate (Millipore). Scrapie cell assay protocol was adapted from Bian et al. 2010 and is described in Hay et al. 2022 [27,86]. Primary antibody Sha31 (Cayman Chemical Company) diluted 1:5000 in TBST (Tris-Buffered Saline with Triton-X) was incubated overnight at 4° C. Secondary antibody, AP-α-Mouse IgG (Southern Biotechnology Associates, Birmingham, AL) was diluted 1: 5000 in TBST. Plates were scanned with a ImmunoSpot S6-V analyzer (Cellular Technology Ltd, Shaker Heights, OH), and determined spot numbers using ImmunoSpot5 software (Cellular Technology Ltd, Shaker Heights, OH).

### Brain preparation and inoculation

Brain homogenates were obtained and prepared as described previously [36]. Mice were inoculated at 6 weeks of age while under anesthesia with 30·l of 1% Rocky Mountain Laboratories (RML) strain of mouse-adapted scrapie brain homogenate or normal brain homogenate (NBH) for mock-infected animals. Inoculum was prepared in PBS containing 1% PSN.

### Behavioral analyses and clinical scoring

Mice were individually caged at the start of behavioral analysis until euthanasia. Nest building activity was evaluated weekly by placing three fresh napkins in the cage. 48 hours later, the position and structure of the napkins was evaluated, as described previously [36]. Nests were evaluated beginning at 13 weeks post-infection (wpi) until euthanasia. Burrowing was analyzed biweekly, as described previously [36], beginning at week 12 and performed every other week, then performed weekly beginning at 16 wpi, for the duration of the study. Clinical signs and weight were evaluated beginning at 13 wpi and continued weekly until mice were euthanized, using previously described scoring methods [36]. Clinical signs and weight monitoring were increased to twice weekly once mice began showing clinical scores of 5 or more. Mice were euthanized when they reached a total score of 10 for any combination of signs. Mice inoculated with NBH were used as controls for behavioral analyses and clinical signs. Infected Cx3Cr1Cre-IKKflox mice succumbed to disease around 17 wpi (*n* = 9). For direct comparison of tissue, both RML-infected (*n* = 6) and NBH-treated C57Bl6/J (*n* = 5), and NBH-treated Cx3Cr1Cre-IKKflox mice (*n* = 4), were euthanized at 17 wpi, despite the non-transgenic wild-type C57Bl6/J and NBH-treated mice not displaying significant clinical signs. Additionally, another cohort of RML-infected C57Bl6/J mice was taken to clinical stages (*n = *7).

### Brain hisbtology

Animals were deeply anaesthetized with isoflurane before euthanasia by decapitation. Brains were removed and the left hemisphere was frozen at -80° C prior to homogenization. The right hemisphere was fixed in 10% neutral buffered formalin for 72 hours. Tissue was embedded in paraffin and 5 μm sections were cut for staining.

#### Hematoxylin and eosin staining.

Hematoxylin and eosin staining of paraffinized brains was performed on the right hemisphere, as described previously [36]. Vacuoles in the frontal cortex, hippocampus, thalamus and cerebellum were assessed for quantity and size and scored from 0 (no pathology) to 5 (significant pathology) by three blinded pathologists and averaged.

#### Immunohistochemistry.

Immunohistochemical staining of paraffinized brains was performed on the right hemisphere, as described previously [36]. Iba1 (Abcam) was used at a 1:400 dilution, GFAP (Dako) was used at a 1:400 dilution, and NeuN (Cell Signaling) was used at a 1:250 dilution. 40x representative images and full brain scans were performed with the Olympus BX53. The Olympus CellSens software (v 1.18) was used to count Iba1+ and GFAP+ cells, and NeuN+ cells in the CA1 region of the hippocampus were counted manually. For NeuN counts, outlier identification and removal were performed using a ROUT outlier test (Q = 10%).

#### Immunofluorescence and glial skeletonization.

Immunofluorescence and glial skeletonization of paraffinized brains was performed on the right hemisphere, as described previously [36]. The following primary antibodies were used: Iba1 (Abcam) at a 1:50 dilution, GFAP (Dako) at a 1:250 dilution, S100β (Abcam) at a 1:750 dilution with C3 (Abcam) at a 1:250 dilution. Three animals were randomly selected per group. For each animal, four regions between the dentate gyrus and CA1-CA3 region of the hippocampus were imaged with an Olympus BX63 fluorescence microscope with a motorized stage and Hamamatsu ORCA-flash 4.0 LT CCD camera and an Olympus Xline apochromat 20X (0.8 N.A.) air objective. Exposures for each stain were kept consistent within each channel. Regions of interest (ROI) were selected with Olympus CellSens software to identify S100β^+^ astrocytes. For each region of the hippocampus, a minimum of 9 cells were analyzed. Mean gray intensity of C3 was determined within the ROI of each S100β^+^ cell containing a visible nucleus and normalized to the lowest value. Skeletonization of astrocytes and microglia was performed using IMARIS 9.9.1 using previously described settings [36], with starting point and seed point thresholds adjusted manually.

### Immunoblotting

The left hemispheres were homogenized and protein was quantified with a BCA. Brain homogenates were treated with 2% Sarkosyl, then digested with 100 ·g/ml proteinase K (PK) for 1 hour at 37°C, and 5.8 μg protein was loaded per sample. For undigested brain homogenates, 27 μg per brain were loaded. Blots were blocked in 5% non-fat milk buffer and antibodies were diluted in TBS-T. Brain PrP was analyzed with antibodies Sha31 (Cayman Chemical Company) at a 1:10,000 dilution and 12B2 (Wageningen University, Netherlands) at a 1:5000 dilution. Total brain IKKβ was analyzed with antibody IKKβ D3OC6 (Cell Signaling) at 1:1000 dilution and β-actin (Novus) was used as a control at 1:20,000 dilution. All primary antibodies were incubated overnight at 4°C. Cell lysates were isolated using the protein lysis buffer (50mM Tris, 150mM NaCl, 2mM EDTA, 1mM MgCl2, 100mM NaF, 10% glycerol, 1% Triton X-100, 1% Na deoxycholate, 0.1% SDS and 125mM sucrose) supplemented with Phos-STOP and protease inhibitors (Roche). A BCA Protein Assay kit (Thermo Fisher Scientific) was used to quantify protein concentration of lysates, and 500 ·g protein was digested with 20 ·g/ml PK (Roche) for PrP^Sc^ blots for 1 hour at 37° C. Digestion was terminated with 2mM PMSF and lysates were spun at 40,000 x g for 1 hour at 4° C before being loaded on a gel. For Total PrP, 20 ·g protein was used per sample. Samples were run on a 4–20% acrylamide SDS page gels (BioRad) and transferred onto PVDF blotting paper (MilliPore). All other blots were blocked and incubated with antibodies in 5% bovine serum albumin (Sigma-Aldrich). For PrP, primary antibody Bar-224 (Cayman Chemical Company) was used at 1:1,000 dilution. Loading control GAPDH was incubated at a 1:10,000 dilution (MilliPore). The protein antibody complex was visualized using SuperSignal West Pico PLUS Chemiluminescent Substrate (Thermo Fisher Scientific) and visualized with the BioRad ChemiDoc MP. Quantification of average band intensity for cell lysates was performed using the “measure” function on ImageJ. For brain homogenates, the absolute density of each sample was measured and local background subtracted with ImageQuant TL 10.2 analysis software, Cytiva Life Sciences.

### Statistical analysis

Unless stated otherwise, outlier identification and removal was performed using a ROUT outlier test (Q = 1%). Cleaned data was measured using a T-test for two groups, or a one-way ANOVA with Tukey’s post-hoc analysis for three or more groups. For behavioral and clinical analysis, a two-way repeated measures ANOVA with a Tukey’s post-hoc analysis, OR whatever was performed. For survival curves, a Log-rank (Mantel-Cox) test was performed. A p-value of 0.05 or less was considered significant for all analyses. All figures present mean average + /- standard error of the mean (SEM). Prism (v 9.1.0) was used for all data analysis and graph generation.

## Supporting information

S1 Table Fold change for all NF-κB-related measures comparing RML-infected IKK KO microglia-limited cultures to RML-infected WT mixed glia cultures. (DOCX)

S1 Fig Flow cytometry characterization of GLAST+ and Iba1 + cell numbers in A WT mixed glia cultures and B mixed glia cultures with IKK KO microglia. **C** Western blots characterizing IKKβ, GFAP and Iba1 expression in primary mixed glial cultures from WT mice and mice with IKK KO microglia.
(DOCX)

S2 Fig A Heat map showing magnitude of gene expression for NBH-treated mixed glia (WT NBH), RML-infected mixed glia (WT RML), NBH-treated IKK KO microglia-limited (IKK KO NBH) and RML-infected IKK KO microglia-limited (IKK KO RML) cultures. **B** Volcano plot comparing NF-κB-associated gene expression between RML-infected WT mixed glia culture and IKK KO microglia-limited culture shows the majority of genes are downregulated in the IKK KO microglia-limited culture. Some specific genes are labeled which are of interest in prion disease, or highly up- or downregulated. X-axis intersections: fold change + /- 2. Y axis intersection: *p*-value < 0.05. Samples are composed of 3 biological replicates (individual well of cells) for each group.
(DOCX)

S3 Fig Mice were weighed weekly during the course of infection. Two-way repeated measures ANOVA and post-hoc Tukey test with means, * *p *< 0.05.
(DOCX)

S4 Fig A Morphology of activated Iba1 + microglia and B morphology of reactive GFAP + astrocytes in the hippocampus at terminal stage of RML infection. Scale bar = 50·m.
(DOCX)

S5 Fig Comparisons of Iba1 + microglia in the cortex, hippocampus, thalamus and cerebellum of terminal IKK KO and terminal WT mice. Welch’s t-test, error bars = SEM. Scale bar = 50·m.
(DOCX)

S6 Fig Iba1 + cells in the A cortex, B hippocampus, C thalamus and D cerebellum were counted and compared between mock-infected WT mice (*n* = 5) and mice with IKK KO microglia (*n* = 4) at 17 weeks post infection. Welch’s t-test, error bars = SEM. (DOCX)

S7 Fig Comparisons of GFAP+ astrocytes in the cortex, hippocampus, thalamus and cerebellum of terminal IKK KO and terminal WT mice. Welch’s t-test, error bars = SEM, *p < 0.05. Scale bar = 50·m.
(DOCX)

S8 Fig GFAP+ cells in the A cortex, B hippocampus, C thalamus and D cerebellum were counted and compared between mock-infected WT mice (*n* = 5) and mice with IKK KO microglia (*n* = 4) at 17 weeks post infection. Welch’s t-test, error bars = SEM, **p < 0.01. (DOCX)

S9 Fig Counts of A Neu+ cells in the CA1 region of the hippocampus between mock-infected WT mice and mice with IKK KO microglia at 17 weeks post infection. Vacuole counts in the **B** cortex, **C** hippocampus, **D** thalamus and **E** cerebellum were counted and compared between mock-infected WT mice (*n* = 5) and mice with IKK KO microglia (*n* = 4) at 17 weeks post infection. Welch’s t-test, error bars = SEM.
(DOCX)

S10 Fig A Amino acid sequence of the mouse prion protein and binding sites of antibodies used. **B** Homogenized brains from terminally infected IKK KO mice and wpi-matched infected WT mice were digested with PK and **C** protease-resistant protein signal was measured using 12B2 antibody. Welch’s t-test with mean.
(DOCX)

S11 Fig A Western blot with Sha31 antibody for PK-resistant PrP in terminally infected IKK KO mouse brains and infected WT wpi-matched controls and B densitometry analysis. **C** Western blot for Total PrP in terminally infected IKK KO mouse brains and infected WT wpi-matched controls and **D** densitometry analysis. Welch’s t-test with mean and One-way ANOVA and post-hoc Tukey test with means, *** *p *< 0.001.
(DOCX)

S12 Fig A Western blot of IKKβ in terminally infected brains and B densitometry analysis. One-way ANOVA and post-hoc Tukey test, error bars = SEM, *p < 0.05, **p < 0.01, *** *p *< 0.001.
(DOCX)

S13 Fig Uncropped western blot TIF images. **A** PK-digested PrP in primary mixed glia, Bar224 antibody. **B** Total PrP in primary mixed glia, Bar224 antibody. **C** GAPDH in primary mixed glia (stripped and reprobed from Total PrP blot B.) **D** PK-digested PrP in age matched and terminal brain homogenate, Bar224 antibody. **E** Total PrP in age matched and terminal brain homogenate, Sha31 antibody with **F** β-actin control. Uncropped western blot images from Supplemental Figures. Primary mixed glial cell lysates probed for **G** IKKβ with **H** GAPDH, **I** GFAP with **J** GAPDH and **K** Iba1 with **L** GAPDH. **M** PK-digested PrP in brain homogenate in age matched WT and IKK KO brains, 12B2 antibody. **N** PK-digested PrP and **O** total PrP in age matched WT and IKK KO brains. **P** IKKβ and **Q** β-actin in NBH and terminal RML infected WT and IKK KO brains.
(DOCX)
