# Supplementary material for: Microglia-specific NF-κB signaling is a critical regulator of prion-induced glial inflammation and neuronal loss
Source: PLoS Pathog. 2025 Jun 18;21(6):e1012582. doi: 10.1371/journal.ppat.1012582 (PMC12185024; doi:10.1371/journal.ppat.1012582)
Supplement: S1 Table — (DOCX) [file ppat.1012582.s001.docx]

**Supplemental Table 1. Fold change for all** NF-κB-related measures comparing RML-infected IKK KO microglia-limited cultures to RML-infected WT mixed glia cultures

| **Gene Symbol** | **Fold Δ** | **p Value** |  | **Gene Symbol** | **Fold Δ** | **p Value** |
| --- | --- | --- | --- | --- | --- | --- |
| *Agt* | 0.64 | 0.196041 |  | *Il1b* | 0.03 | 0.000268 |
| *Akt1* | 0.81 | 0.337879 |  | *Il1r1* | 1.62 | 0.003326 |
| *Atf1* | 0.75 | 0.002623 |  | *Irak1* | 1.33 | 0.004616 |
| *Atf2* | 0.95 | 0.666007 |  | *Irak2* | 0.37 | 0.000082 |
| *Bcl10* | 0.79 | 0.008004 |  | *Irf1* | 0.65 | 0.003808 |
| *Bcl2a1a* | 0.01 | 0.000004 |  | *Jun* | 1.08 | 0.342627 |
| *Bcl2l1* | 1.51 | 0.00977 |  | *Lta* | 2.3 | 0.002536 |
| *Bcl3* | 0.17 | 0.000452 |  | *Ltbr* | 0.92 | 0.307473 |
| *Birc3* | 0.24 | 0.000468 |  | *Map3k1* | 0.23 | 0.000359 |
| *Card10* | 1.39 | 0.09038 |  | *Mapk3* | 0.69 | 0.019567 |
| *Card11* | 0.05 | 0.0017 |  | *Myd88* | 0.45 | 0.008061 |
| *Casp1* | 0.3 | 0.000124 |  | *Nfkb1* | 0.45 | 0.000011 |
| *Casp8* | 0.24 | 0.000513 |  | *Nfkb2* | 0.82 | 0.025294 |
| *Ccl2* | 0.43 | 0.018154 |  | *Nfkbia* | 0.54 | 0.015664 |
| *Ccl5* | 0.08 | 4.4E-05 |  | *Nod1* | 0.55 | 0.000286 |
| *Cd27* | 0.39 | 0.035893 |  | *Raf1* | 0.97 | 0.425493 |
| *Cd40* | 0.31 | 0.00789 |  | *Rel* | 0.18 | 0.000345 |
| *Cflar* | 0.66 | 0.003591 |  | *Rela* | 0.77 | 0.015478 |
| *Chuk* | 0.63 | 0.028421 |  | *Relb* | 0.48 | 0.003334 |
| *Crebbp* | 1.42 | 0.06739 |  | *Ripk1* | 0.41 | 0.001633 |
| *Csf1* | 3.25 | 0.002318 |  | *Ripk2* | 0.73 | 0.055235 |
| *Csf2* | 0.58 | 0.392593 |  | *Slc20a1* | 1.74 | 0.001436 |
| *Csf3* | 1.18 | 0.525584 |  | *Smad3* | 0.96 | 0.408529 |
| *Egfr* | 3.95 | 0.002812 |  | *Stat1* | 0.52 | 0.000025 |
| *Egr1* | 0.44 | 0.035673 |  | *Tbk1* | 0.61 | 0.001387 |
| *Eif2ak2* | 0.98 | 0.789113 |  | *Tlr1* | 0.01 | 0.002771 |
| *Elk1* | 1.17 | 0.101562 |  | *Tlr2* | 0.05 | 0.000119 |
| *F2r* | 2.4 | 0.000501 |  | *Tlr3* | 0.87 | 0.48881 |
| *Fadd* | 0.94 | 0.477514 |  | *Tlr4* | 0.29 | 0.000413 |
| *Fos* | 0.7 | 0.186652 |  | *Tlr6* | 0.09 | 0.000105 |
| *Hmox1* | 0.46 | 0.000644 |  | *Tlr9* | 0.01 | 8E-06 |
| *Icam1* | 0.53 | 0.000181 |  | *Tnf* | 0.01 | 0.000212 |
| *Ikbkb* | 0.54 | 0.001293 |  | *Tnfaip3* | 0.24 | 0.003867 |
| **Gene Symbol** | **Fold Δ** | **p Value** |  | **Gene Symbol** | **Fold Δ** | **p Value** |
| *Ikbke* | 0.13 | 0.004161 |  | *Tnfrsf10b* | 2.48 | 0.002526 |
| *Ikbkg* | 1.4 | 0.047339 |  | *Tnfrsf1a* | 1.24 | 0.042934 |
| *Il10* | 0.06 | 0.000605 |  | *Tnfrsf1b* | 0.09 | 0.00024 |
| *Il1a* | 0.02 | 0.000996 |  | *Tnfsf10* | 0.19 | 0.004815 |
| *Tnfsf14* | 0.09 | 0.001655 |  | *Traf3* | 1.28 | 0.013968 |
| *Tollip* | 1.43 | 0.004736 |  | *Traf5* | 0.4 | 0.000134 |
| *Tradd* | 0.85 | 0.005738 |  | *Traf6* | 0.73 | 0.023443 |
| *Traf2* | 0.76 | 0.004957 |  | *Zap70* | 0.93 | 0.628275 |
