# Supplementary material for: Microglia-specific NF-κB signaling is a critical regulator of prion-induced glial inflammation and neuronal loss
Source: PLoS Pathog. 2025 Jun 18;21(6):e1012582. doi: 10.1371/journal.ppat.1012582 (PMC12185024; doi:10.1371/journal.ppat.1012582)
Supplement: S1 Fig — C Western blots characterizing IKKβ, GFAP and Iba1 expression in primary mixed glial cultures from WT mice and mice with IKK KO microglia. (DOCX) [file ppat.1012582.s002.docx]

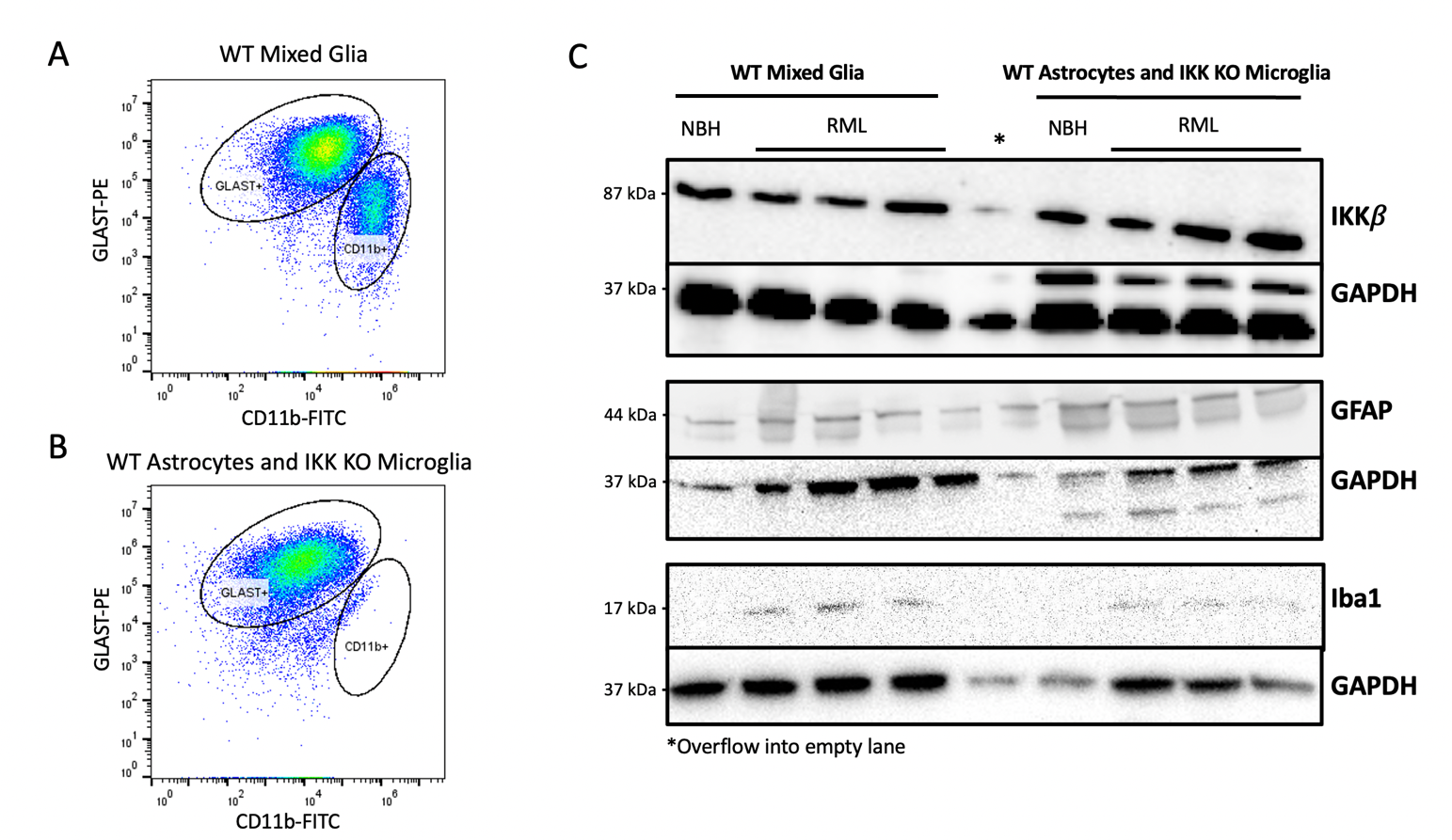


**Supplemental Figure 1.** Flow cytometry characterization of GLAST+ and Iba1+ cell numbers in **A** WT mixed glia cultures and **B** mixed glia cultures with IKK KO microglia. **C** Western blots characterizing IKKβ, GFAP and Iba1 expression in primary mixed glial cultures from WT mice and mice with IKK KO microglia.
