# Supplementary material for: Microglia-specific NF-κB signaling is a critical regulator of prion-induced glial inflammation and neuronal loss
Source: PLoS Pathog. 2025 Jun 18;21(6):e1012582. doi: 10.1371/journal.ppat.1012582 (PMC12185024; doi:10.1371/journal.ppat.1012582)
Supplement: S2 Fig — B Volcano plot comparing NF-κB-associated gene expression between RML-infected WT mixed glia culture and IKK KO microglia-limited culture shows the majority of genes are downregulated in the IKK KO microglia-limited culture. Some specific genes are labeled which are of interest in prion disease, or highly up- or downregulated. X-axis intersections: fold change + /- 2. Y axis intersection: p-value < 0.05. Samples are composed of 3 biological replicates (individual well of cells) for each group. (DOCX) [file ppat.1012582.s003.docx]

**
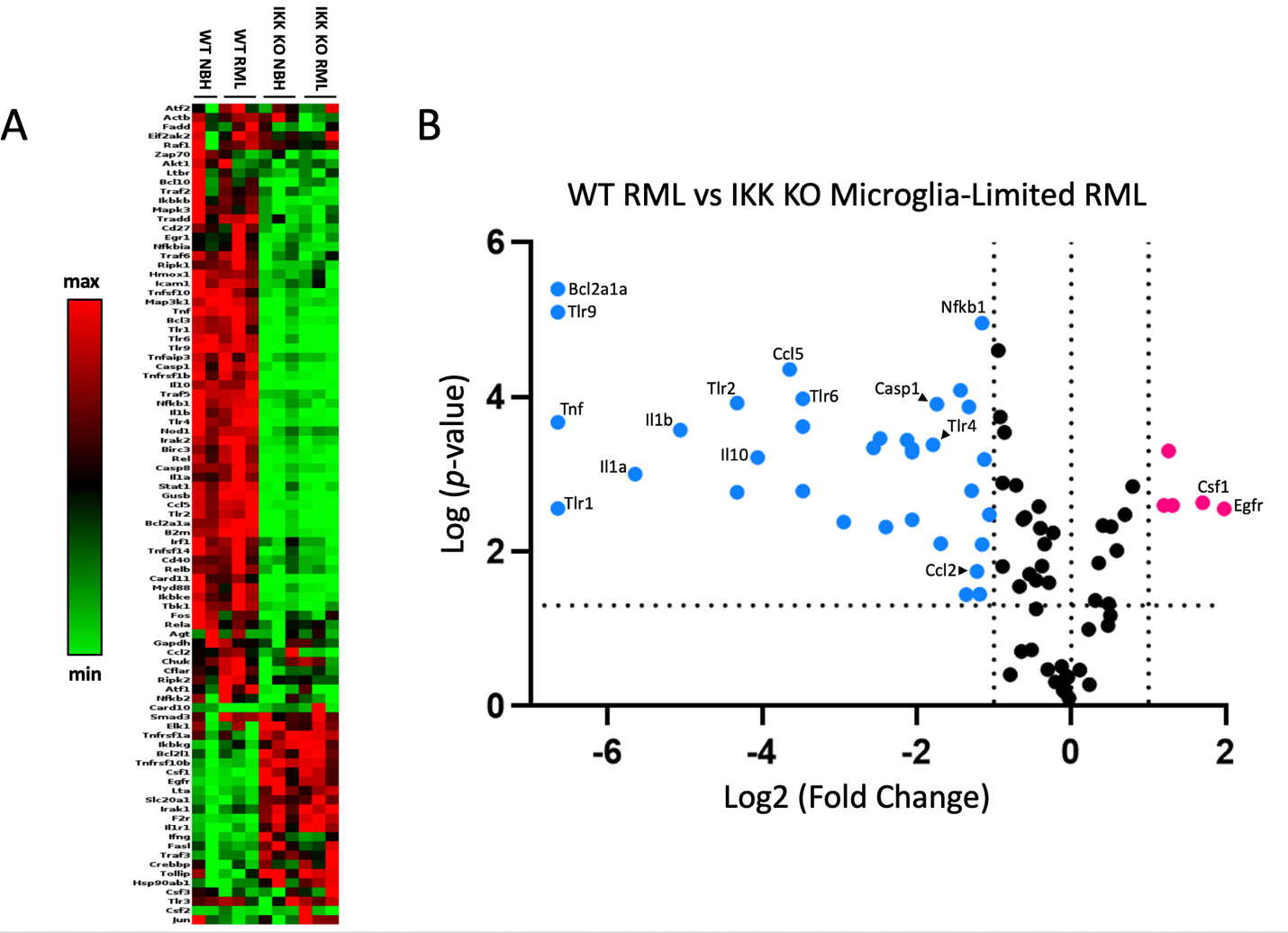
**

**Supplemental Figure 2. A** Heat map showing magnitude of gene expression for NBH-treated mixed glia (WT NBH), RML-infected mixed glia (WT RML), NBH-treated IKK KO microglia-limited (IKK KO NBH) and RML-infected IKK KO microglia-limited (IKK KO RML) cultures. **B** Volcano plot comparing NF-κB-associated gene expression between RML-infected WT mixed glia culture and IKK KO microglia-limited culture shows the majority of genes are downregulated in the IKK KO microglia-limited culture. Some specific genes are labeled which are of interest in prion disease, or highly up- or downregulated. X-axis intersections: fold change +/- 2. Y axis intersection: *p*-value < 0.05. Samples are composed of 3 biological replicates (individual well of cells) for each group.
