# Supplementary material for: Microglia-specific NF-κB signaling is a critical regulator of prion-induced glial inflammation and neuronal loss
Source: PLoS Pathog. 2025 Jun 18;21(6):e1012582. doi: 10.1371/journal.ppat.1012582 (PMC12185024; doi:10.1371/journal.ppat.1012582)
Supplement: S3 Fig — Two-way repeated measures ANOVA and post-hoc Tukey test with means, * p < 0.05. (DOCX) [file ppat.1012582.s004.docx]

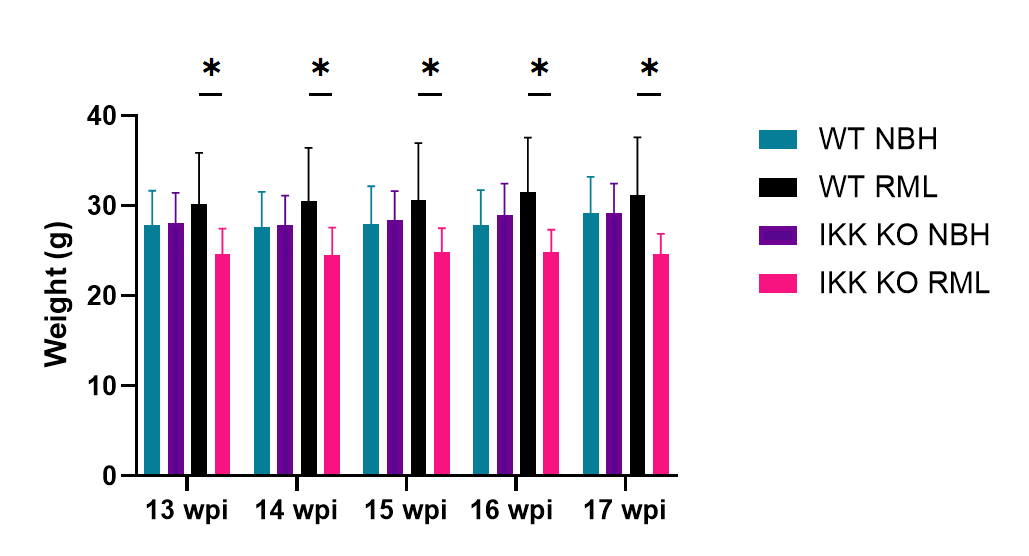


**Supplemental Figure 3.** Mice were weighed weekly during the course of infection. Two-way repeated measures ANOVA and post-hoc Tukey test with means, * *p* < 0.05.
