# Supplementary material for: Microglia-specific NF-κB signaling is a critical regulator of prion-induced glial inflammation and neuronal loss
Source: PLoS Pathog. 2025 Jun 18;21(6):e1012582. doi: 10.1371/journal.ppat.1012582 (PMC12185024; doi:10.1371/journal.ppat.1012582)
Supplement: S4 Fig — Scale bar = 50·m. (DOCX) [file ppat.1012582.s005.docx]

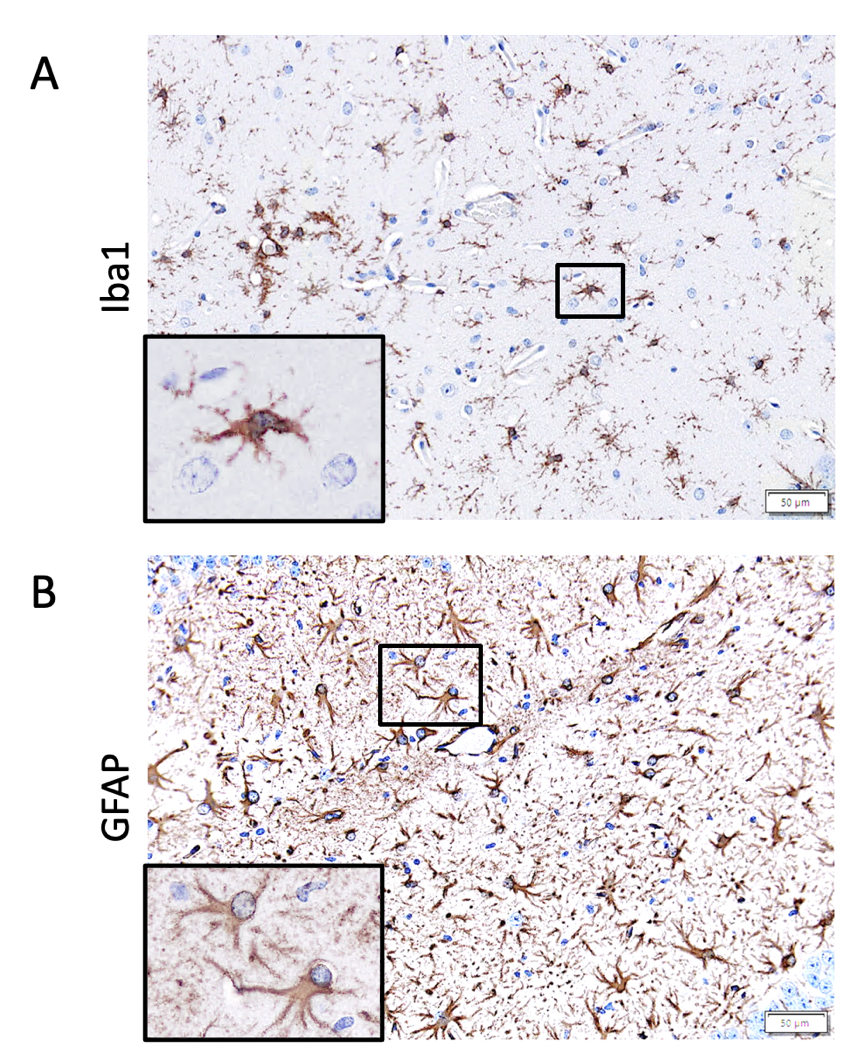


**Supplemental Figure 4. A** Morphology of activated Iba1+ microglia and **B** morphology of reactive GFAP+ astrocytes in the hippocampus at terminal stage of RML infection. Scale bar = 50μm
