# Supplementary material for: Microglia-specific NF-κB signaling is a critical regulator of prion-induced glial inflammation and neuronal loss
Source: PLoS Pathog. 2025 Jun 18;21(6):e1012582. doi: 10.1371/journal.ppat.1012582 (PMC12185024; doi:10.1371/journal.ppat.1012582)
Supplement: S5 Fig — Welch’s t-test, error bars = SEM. Scale bar = 50·m. (DOCX) [file ppat.1012582.s006.docx]

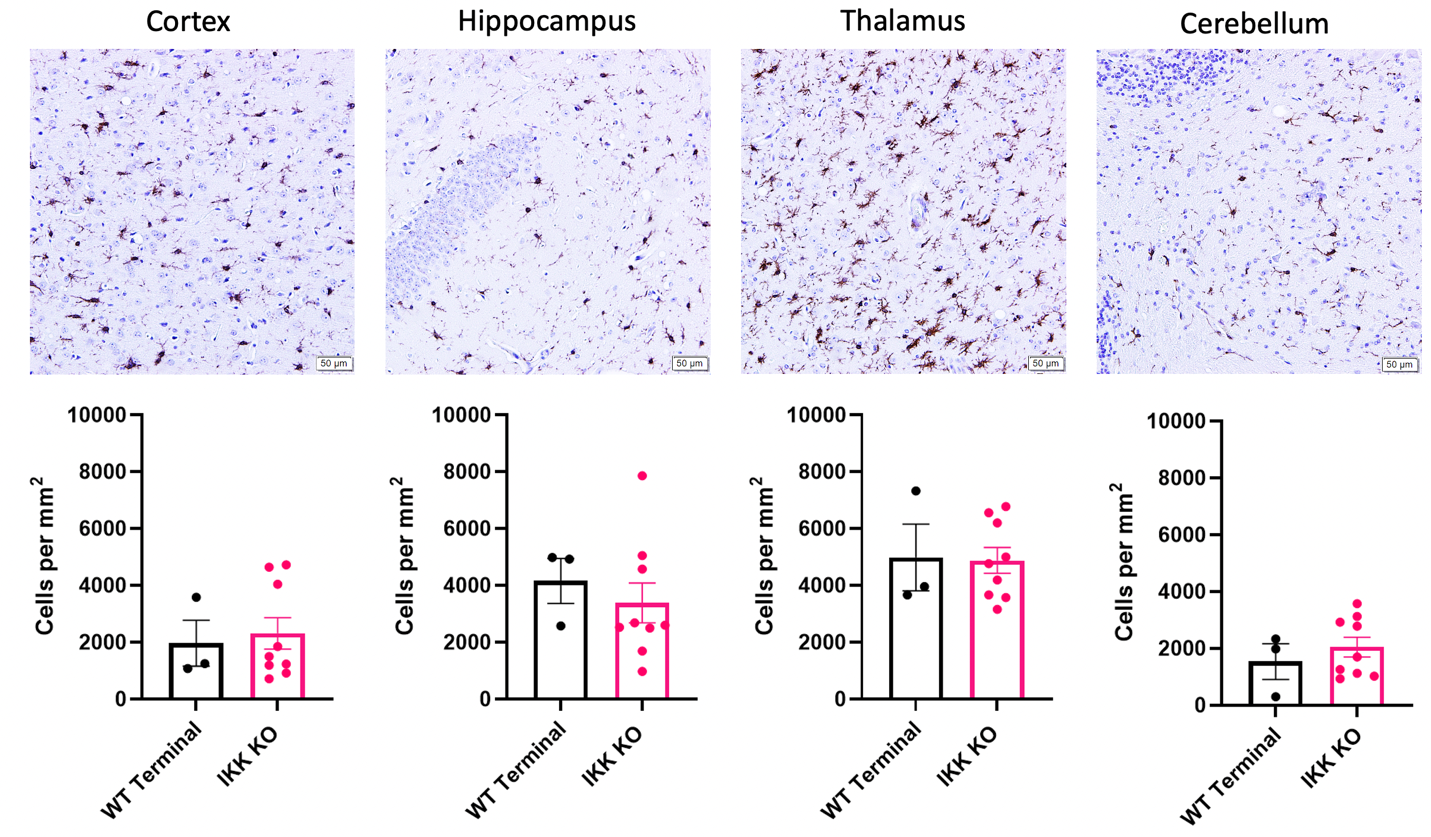


**Supplemental Figure 5.** Comparisons of Iba1+ microglia in the cortex, hippocampus, thalamus and cerebellum of terminal IKK KO and terminal WT mice. Welch’s t-test, error bars = SEM. Scale bar = 50μm
