# Supplementary material for: Microglia-specific NF-κB signaling is a critical regulator of prion-induced glial inflammation and neuronal loss
Source: PLoS Pathog. 2025 Jun 18;21(6):e1012582. doi: 10.1371/journal.ppat.1012582 (PMC12185024; doi:10.1371/journal.ppat.1012582)
Supplement: S6 Fig — (DOCX) [file ppat.1012582.s007.docx]

**
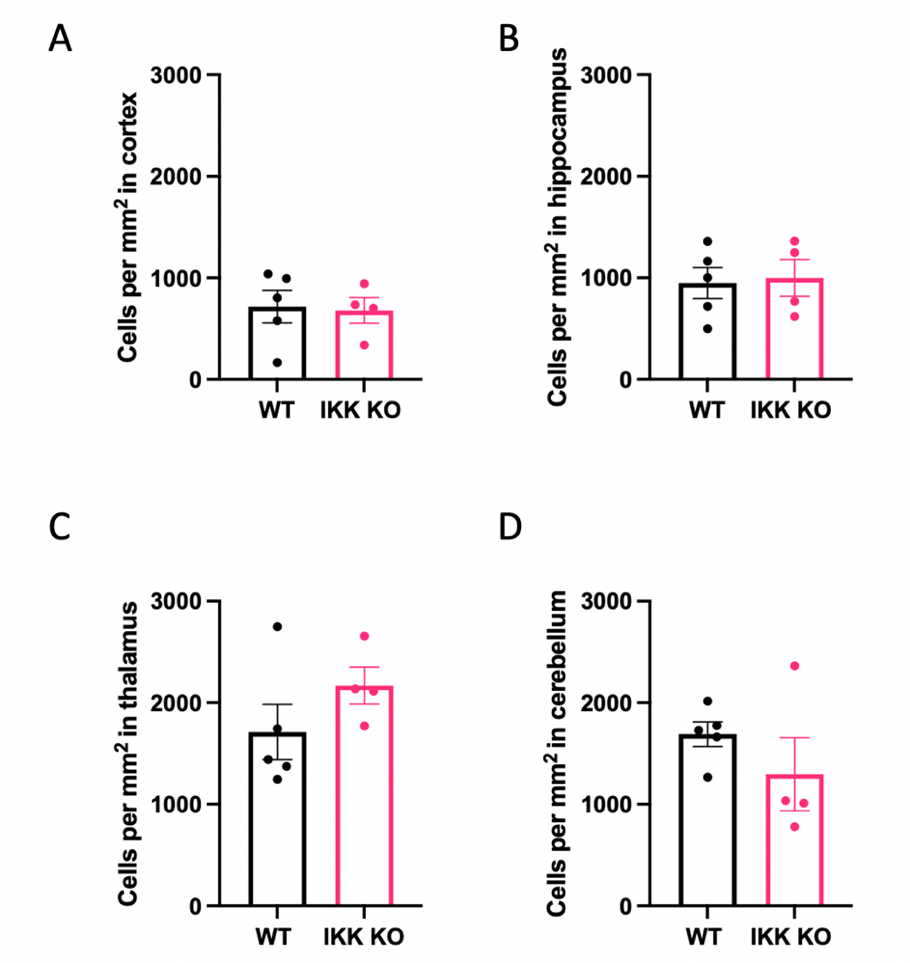
**

**Supplemental Figure 6.** Iba1+ cells in the **A** cortex, **B** hippocampus, **C** thalamus and **D** cerebellum were counted and compared between mock-infected WT mice (*n*=5) and mice with IKK KO microglia (*n*=4) at 17 weeks post infection. Welch’s t-test, error bars = SEM.
