# Supplementary material for: Microglia-specific NF-κB signaling is a critical regulator of prion-induced glial inflammation and neuronal loss
Source: PLoS Pathog. 2025 Jun 18;21(6):e1012582. doi: 10.1371/journal.ppat.1012582 (PMC12185024; doi:10.1371/journal.ppat.1012582)
Supplement: S9 Fig — Vacuole counts in the B cortex, C hippocampus, D thalamus and E cerebellum were counted and compared between mock-infected WT mice (n = 5) and mice with IKK KO microglia (n = 4) at 17 weeks post infection. Welch’s t-test, error bars = SEM. (DOCX) [file ppat.1012582.s010.docx]

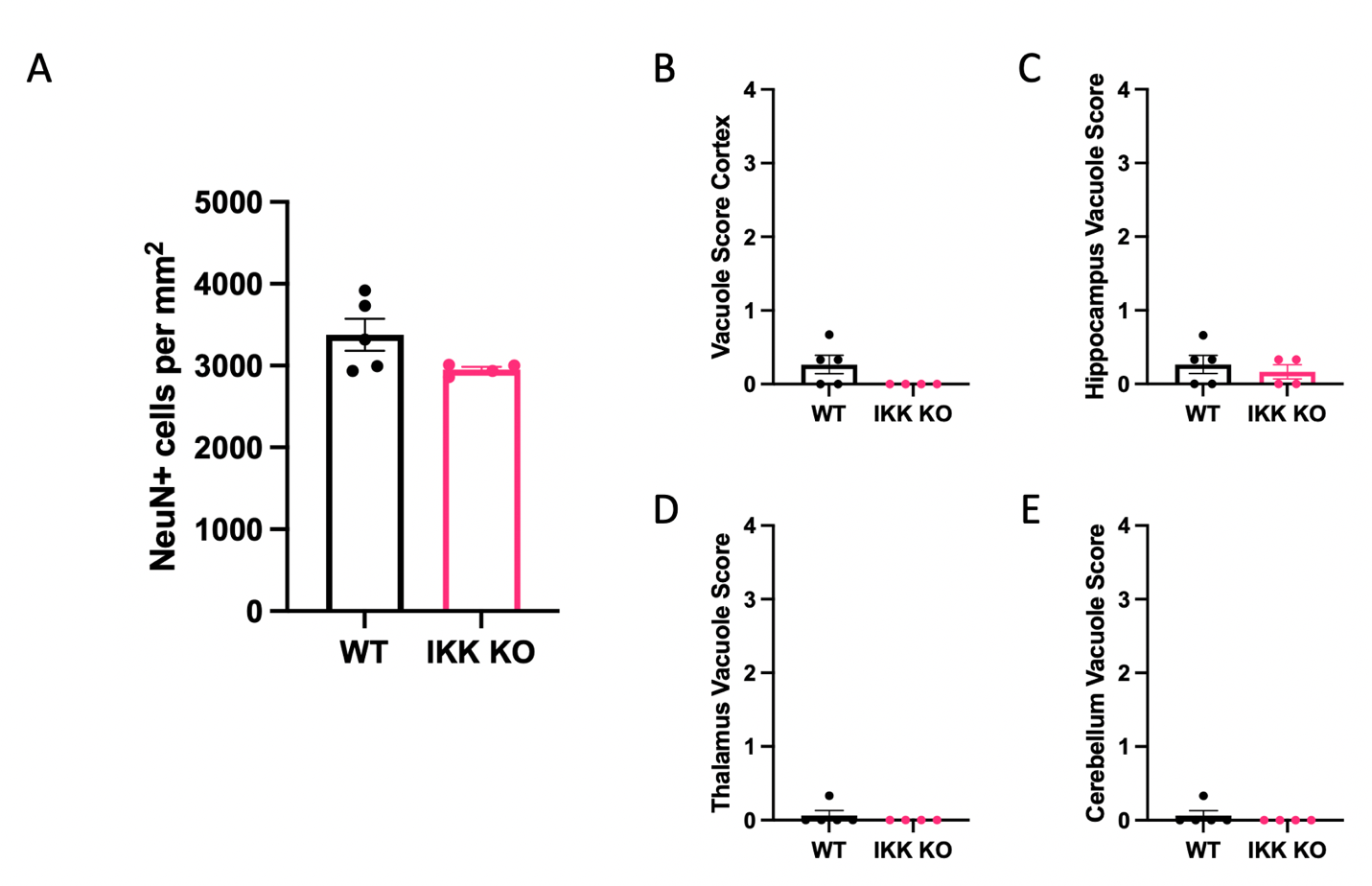


**Supplemental Figure 9.** Counts of **A** Neu+ cells in the CA1 region of the hippocampus between mock-infected WT mice and mice with IKK KO microglia at 17 weeks post infection. Vacuole counts in the **B** cortex, **C** hippocampus, **D** thalamus and **E** cerebellum were counted and compared between mock-infected WT mice (*n*=5) and mice with IKK KO microglia (*n*=4) at 17 weeks post infection. Welch’s t-test, error bars = SEM.
