# Supplementary material for: Microglia-specific NF-κB signaling is a critical regulator of prion-induced glial inflammation and neuronal loss
Source: PLoS Pathog. 2025 Jun 18;21(6):e1012582. doi: 10.1371/journal.ppat.1012582 (PMC12185024; doi:10.1371/journal.ppat.1012582)
Supplement: S10 Fig — B Homogenized brains from terminally infected IKK KO mice and wpi-matched infected WT mice were digested with PK and C protease-resistant protein signal was measured using 12B2 antibody. Welch’s t-test with mean. (DOCX) [file ppat.1012582.s011.docx]

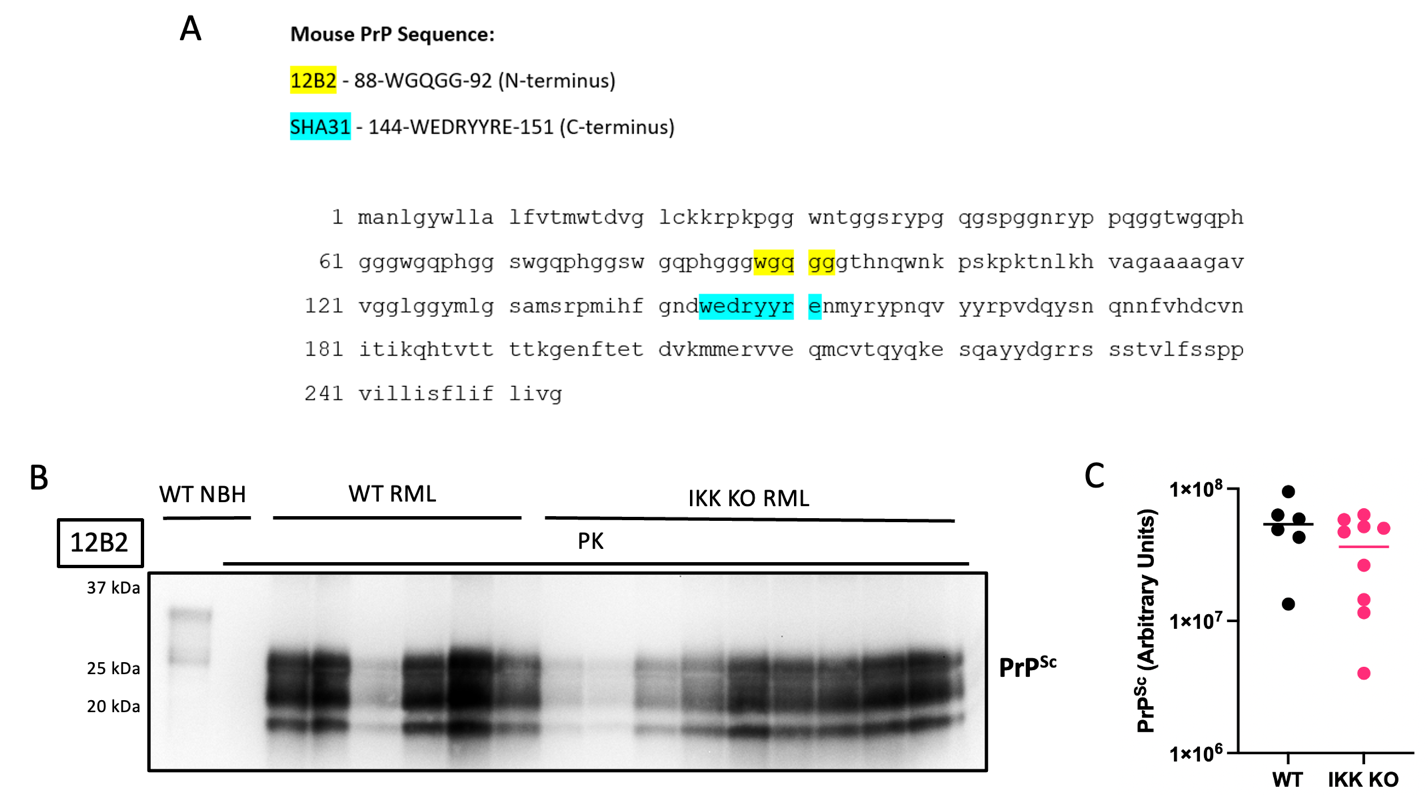


**Supplemental Figure 10. A** Amino acid sequence of the mouse prion protein and binding sites of antibodies used. **B** Homogenized brains from terminally infected IKK KO mice and wpi-matched infected WT mice were digested with PK and **C** protease-resistant protein signal was measured using 12B2 antibody. Welch’s t-test with mean.
