# Supplementary material for: Microglia-specific NF-κB signaling is a critical regulator of prion-induced glial inflammation and neuronal loss
Source: PLoS Pathog. 2025 Jun 18;21(6):e1012582. doi: 10.1371/journal.ppat.1012582 (PMC12185024; doi:10.1371/journal.ppat.1012582)
Supplement: S11 Fig — C Western blot for Total PrP in terminally infected IKK KO mouse brains and infected WT wpi-matched controls and D densitometry analysis. Welch’s t-test with mean and One-way ANOVA and post-hoc Tukey test with means, *** p < 0.001. (DOCX) [file ppat.1012582.s012.docx]

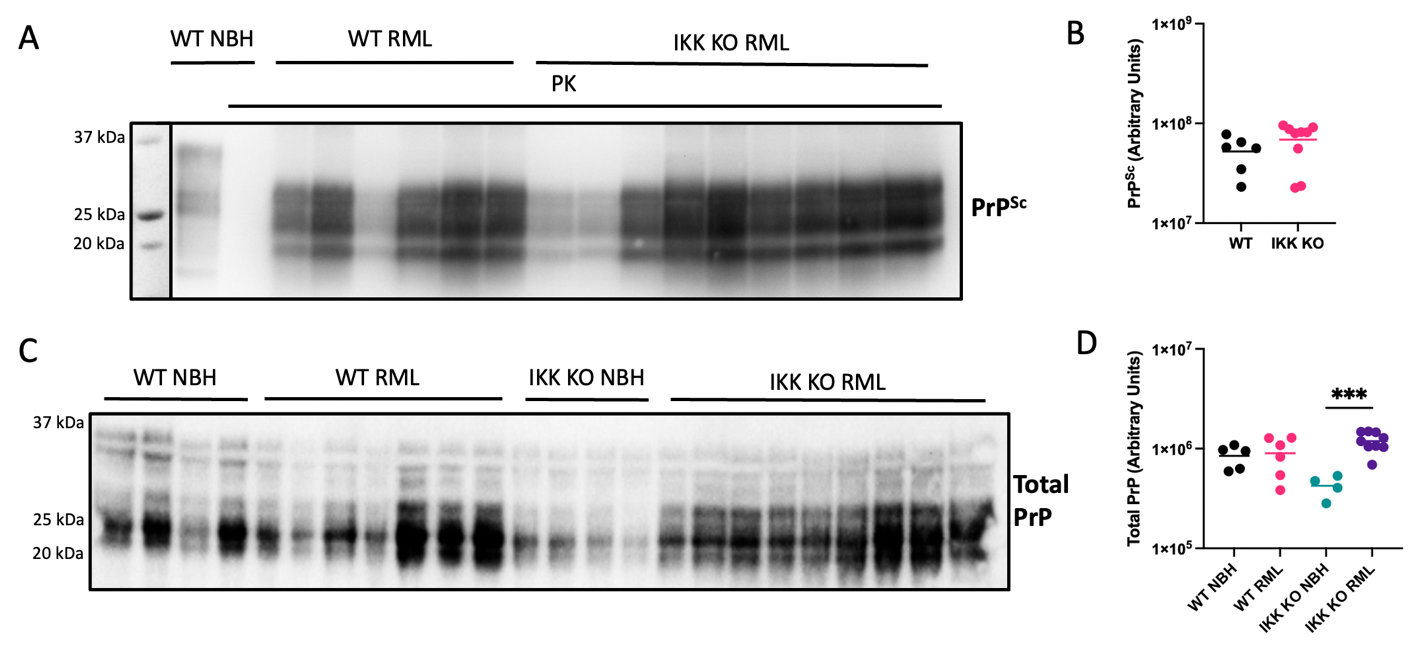


**Supplemental Figure 11. A** Western blot with Sha31 antibody for PK-resistant PrP in terminally infected IKK KO mouse brains and infected WT wpi-matched controls and **B** densitometry analysis. **C** Western blot for Total PrP in terminally infected IKK KO mouse brains and infected WT wpi-matched controls and **D** densitometry analysis. Welch’s t-test with mean and One-way ANOVA and post-hoc Tukey test with means, *** *p* < 0.001.
