# Supplementary material for: Microglia-specific NF-κB signaling is a critical regulator of prion-induced glial inflammation and neuronal loss
Source: PLoS Pathog. 2025 Jun 18;21(6):e1012582. doi: 10.1371/journal.ppat.1012582 (PMC12185024; doi:10.1371/journal.ppat.1012582)
Supplement: S12 Fig — One-way ANOVA and post-hoc Tukey test, error bars = SEM, *p < 0.05, **p < 0.01, *** p < 0.001. (DOCX) [file ppat.1012582.s013.docx]

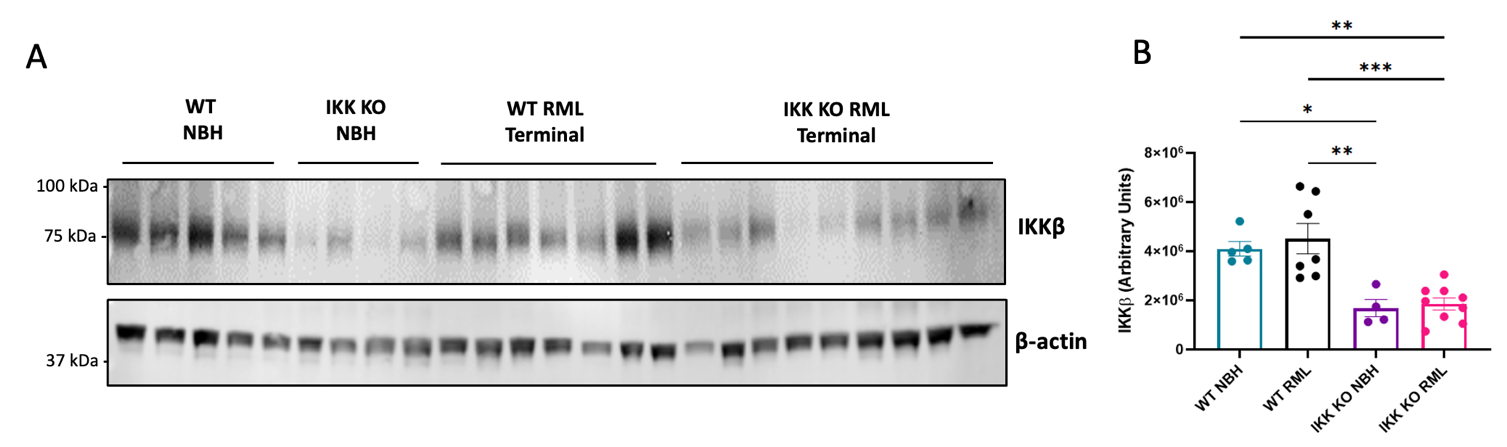


**Supplemental Figure 12. A** Western blot of IKKβ in terminally infected brains and **B** densitometry analysis. One-way ANOVA and post-hoc Tukey test, error bars = SEM, **p* < 0.05, ***p* < 0.01, *** *p* < 0.001.
