# Supplementary material for: Microglia-specific NF-κB signaling is a critical regulator of prion-induced glial inflammation and neuronal loss
Source: PLoS Pathog. 2025 Jun 18;21(6):e1012582. doi: 10.1371/journal.ppat.1012582 (PMC12185024; doi:10.1371/journal.ppat.1012582)
Supplement: S13 Fig — A PK-digested PrP in primary mixed glia, Bar224 antibody. B Total PrP in primary mixed glia, Bar224 antibody. C GAPDH in primary mixed glia (stripped and reprobed from Total PrP blot B.) D PK-digested PrP in age matched and terminal brain homogenate, Bar224 antibody. E Total PrP in age matched and terminal brain homogenate, Sha31 antibody with F β-actin control. Uncropped western blot images from Supplemental Figures. Primary mixed glial cell lysates probed for G IKKβ with H GAPDH, I GFAP with J GAPDH and K Iba1 with L GAPDH. M PK-digested PrP in brain homogenate in age matched WT and IKK KO brains, 12B2 antibody. N PK-digested PrP and O total PrP in age matched WT and IKK KO brains. P IKKβ and Q β-actin in NBH and terminal RML infected WT and IKK KO brains. (DOCX) [file ppat.1012582.s014.docx]

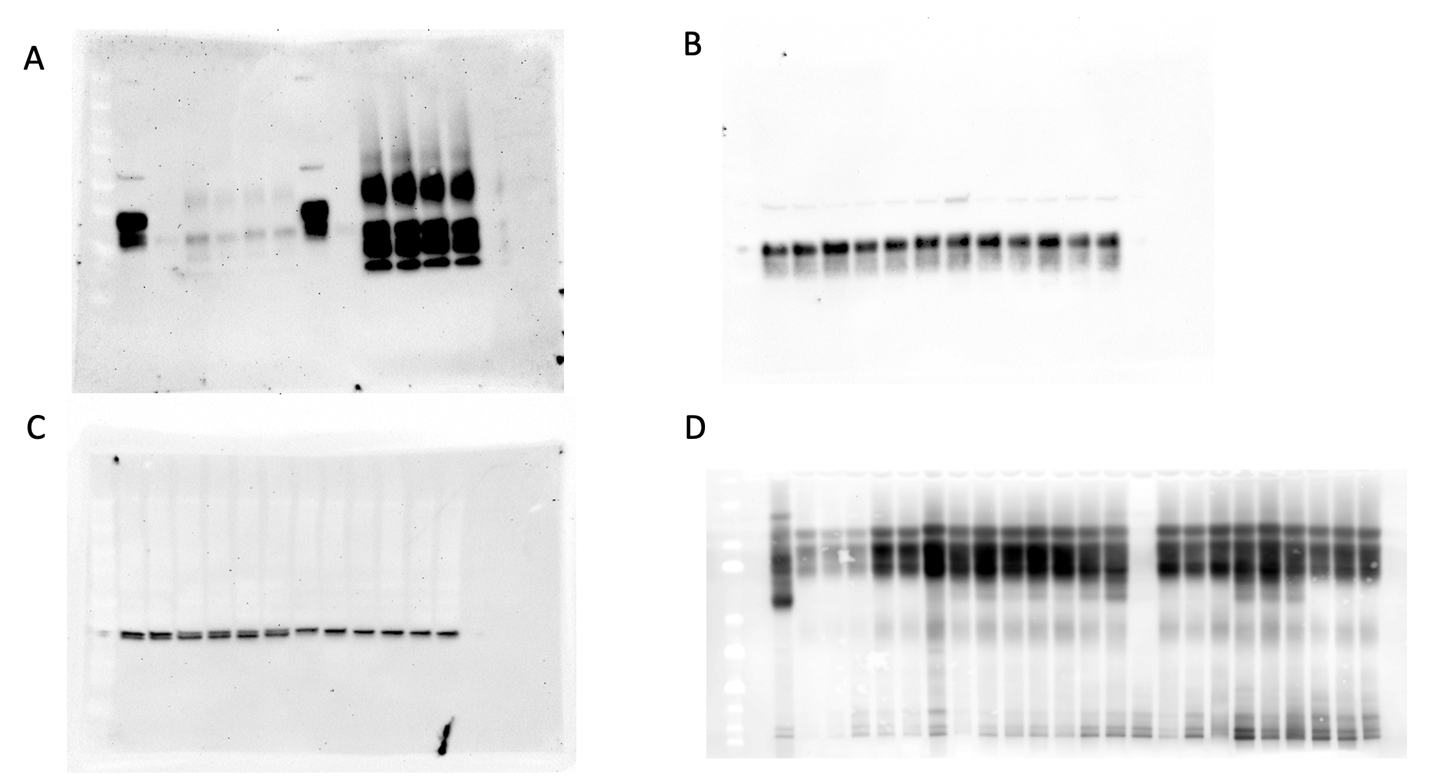


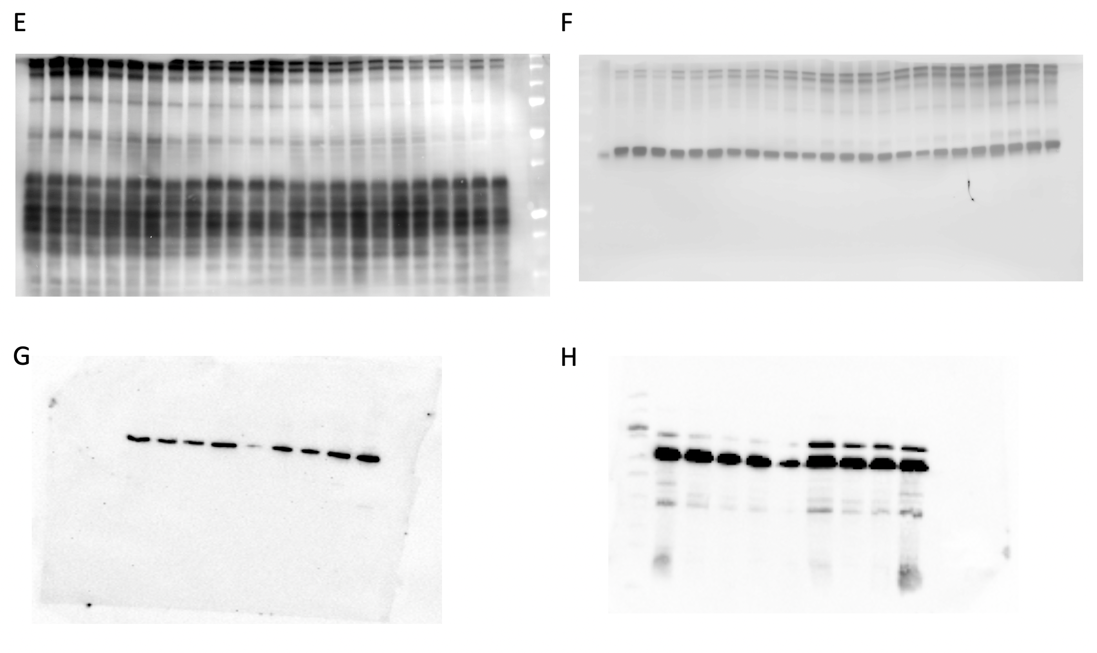


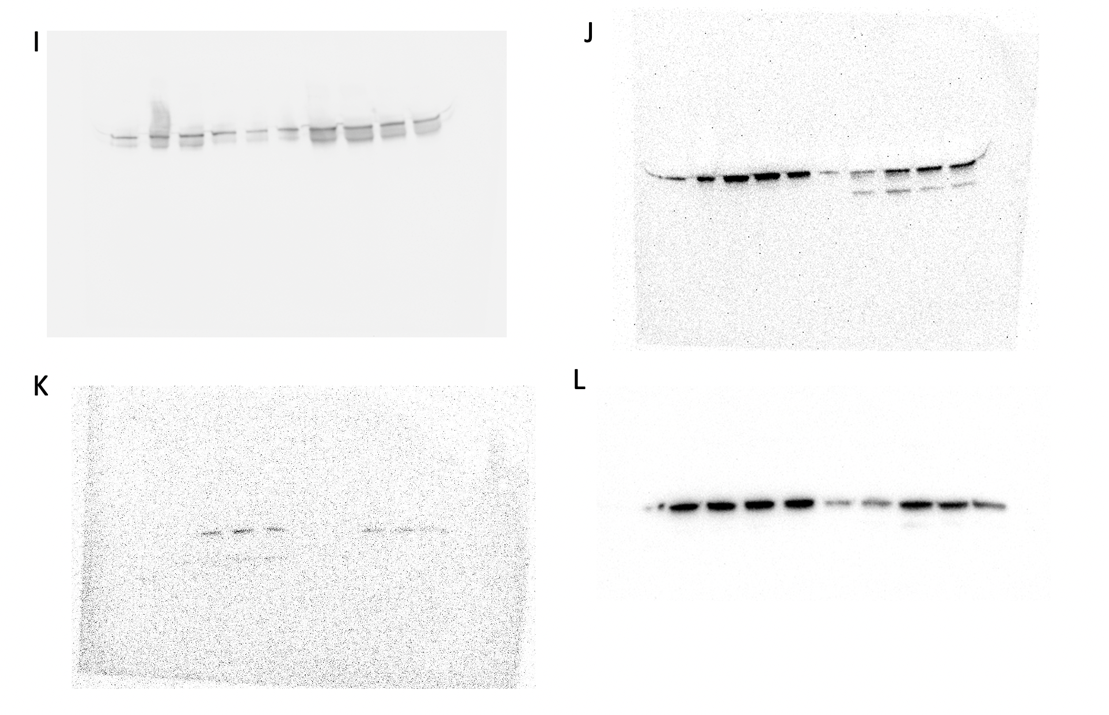


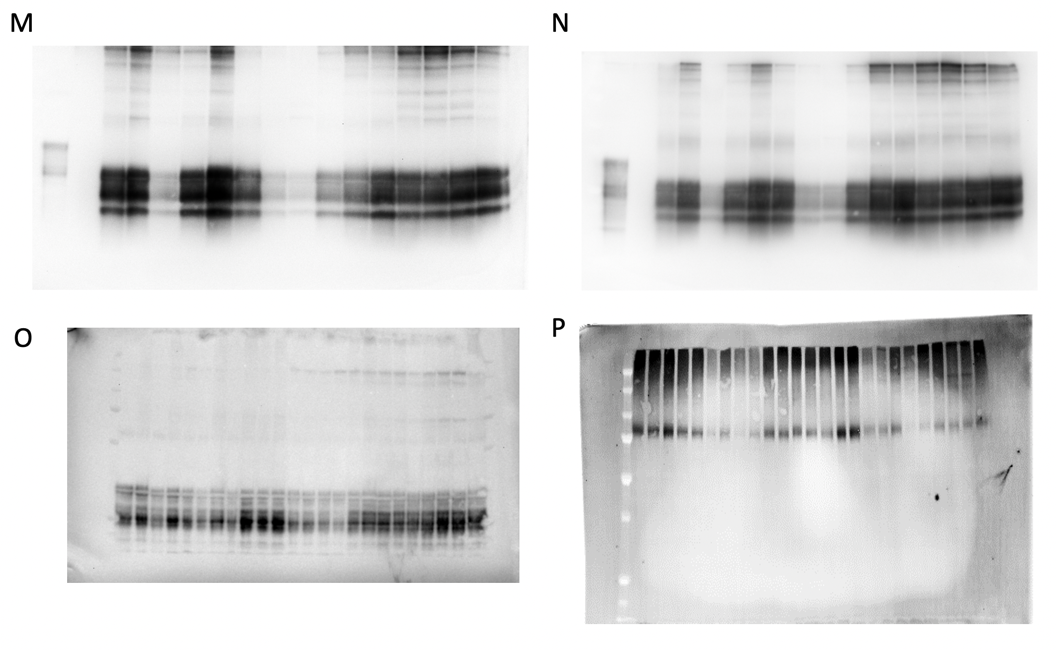


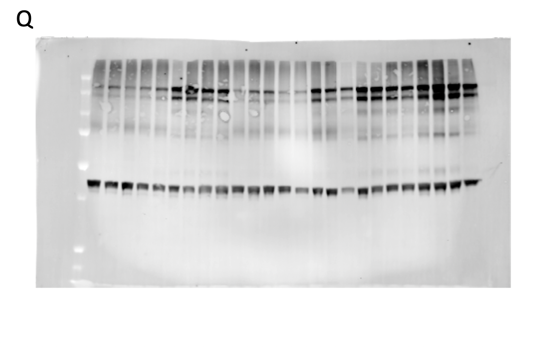


**Supplemental Figure 13.** Uncropped western blot TIF images. **A** PK-digested PrP in primary mixed glia, Bar224 antibody. **B** Total PrP in primary mixed glia, Bar224 antibody. **C** GAPDH in primary mixed glia (stripped and reprobed from Total PrP blot B.) **D** PK-digested PrP in age matched and terminal brain homogenate, Bar224 antibody. **E** Total PrP in age matched and terminal brain homogenate, Sha31 antibody with **F** β-actin control. Uncropped western blot images from Supplemental Figures. Primary mixed glial cell lysates probed for **G** IKKβ with **H** GAPDH, **I** GFAP with **J** GAPDH and **K** Iba1 with **L** GAPDH. **M** PK-digested PrP in brain homogenate in age matched WT and IKK KO brains, 12B2 antibody. **N** PK-digested PrP and **O** total PrP in age matched WT and IKK KO brains. **P** IKKβ and **Q** β-actin in NBH and terminal RML infected WT and IKK KO brains.
